# The impact of COVID-19 on young people’s mental health, wellbeing and routine from a European perspective: A co-produced qualitative systematic review

**DOI:** 10.1371/journal.pone.0299547

**Published:** 2024-03-20

**Authors:** Lindsay H. Dewa, Lily Roberts, Elizabeth Choong, Caroline Crandell, Ola Demkowicz, Emma Ashworth, Catia Branquinho, Steph Scott

**Affiliations:** 1 NIHR Patient Safety Translational Research Centre, Institute of Global Health Innovation, Imperial College London, London, United Kingdom; 2 School of Public Health, Imperial College London, London, United Kingdom; 3 Centre for Health Policy, Institute of Global Health Innovation, Imperial College London, London, United Kingdom; 4 Liggins Institute, University of Auckland Waipapa Taumata Rau, Auckland, New Zealand; 5 Manchester Institute of Education, The University of Manchester, Manchester, United Kingdom; 6 School of Psychology, Liverpool John Moores University, Liverpool, United Kingdom; 7 Environmental Health Institute, Medicine Faculty, University of Lisbon, Lisbon, Portugal; 8 Newcastle Population Health Sciences Institute, Faculty of Medical Sciences, University of Newcastle, Newcastle upon Tyne, United Kingdom; Public Library of Science, UNITED KINGDOM

## Abstract

**Background:**

The impact of the Covid-19 pandemic on young people’s (YP) mental health has been mixed. Systematic reviews to date have focused predominantly on quantitative studies and lacked involvement from YP with lived experience of mental health difficulties. Therefore, our primary aim was to conduct a qualitative systematic review to examine the perceived impact of the Covid-19 pandemic on YP’s (aged 10–24) mental health and wellbeing across Europe.

**Methods and findings:**

We searched MEDLINE, PsycINFO, Embase, Web of Science, MEDRXIV, OSF preprints, Google, and voluntary sector websites for studies published from 1^st^ January 2020 to 15^th^ November 2022. European studies were included if they reported qualitative data that could be extracted on YP’s (aged 10–24) own perspectives of their experiences of Covid-19 and related disruptions to their mental health and wellbeing. Screening, data extraction and appraisal was conducted independently in duplicate by researchers and YP with lived experience of mental health difficulties (co-researchers). Confidence was assessed using the Confidence in the Evidence from Reviews of Qualitative Research (CERQual) approach. We co-produced an adapted narrative thematic synthesis with co-researchers. This study is registered with PROSPERO, CRD42021251578. We found 82 publications and included 77 unique studies in our narrative synthesis. Most studies were from the UK (n = 50; 65%); and generated data during the first Covid-19 wave (March-May 2020; n = 33; 43%). Across the 79,491 participants, views, and experiences of YP minoritised by ethnicity and sexual orientation, and from marginalised or vulnerable YP were limited. Five synthesised themes were identified: negative impact of pandemic information and restrictions on wellbeing; education and learning on wellbeing; social connection to prevent loneliness and disconnection; emotional, lifestyle and behavioural changes; and mental health support. YP’s mental health and wellbeing across Europe were reported to have fluctuated during the pandemic. Challenges were similar but coping strategies to manage the impact of these challenges on mental health varied across person, study, and country. Short-term impacts were related to the consequences of changing restrictions on social connection, day-to-day lifestyle, and education set-up. However, YP identified potential issues in these areas going forward, and therefore stressed the importance of ongoing long-term support in education, learning and mental health post-Covid-19.

**Conclusions:**

Our findings map onto the complex picture seen from quantitative systematic reviews regarding the impact of Covid-19 on YP’s mental health. The comparatively little qualitative data found in our review means there is an urgent need for more high-quality qualitative research outside of the UK and/or about the experiences of minoritised groups to ensure all voices are heard and everyone is getting the support they need following the pandemic. YP’s voices need to be prioritised in decision-making processes on education, self-care strategies, and mental health and wellbeing, to drive impactful, meaningful policy changes in anticipation of a future systemic crisis.

## Introduction

Adolescence and young adulthood (aged 10–24) represent critical periods of rapid physiological, social, and emotional development. This makes this population group vulnerable to mental health difficulties independent of Covid-19 pandemic [1, 2]. Three-quarters of mental health difficulties including depression, anxiety, and poor psychological wellbeing start before aged 24 [3] and one in six 7–16-year-olds have a probable mental health disorder [4]. The risk of developing these difficulties increases in those with sociodemographic (e.g., lower economic status) [5] and demographic (e.g., minoritised by ethnicity [6], disability [7], or LGBTQ+ status [8]) vulnerabilities. The last decade has seen increased emphasis on awareness campaigns, alongside a drive for education settings to engage in mental health promotion and illness prevention. However, there is limited evidence of their effectiveness [9] alongside significant barriers to accessing mental health services including lengthy waiting times (e.g., National Health Service, United Kingdom) and affordability (e.g., private sector) Europe-wide.

Across Europe, Covid-19 pandemic restrictions brought considerable disruption for young people (YP; aged 10–24), including enforced physical distancing [10], changes to teaching and learning [11, 12], and reduced access to mental health support [13] Quantifying the pandemic’s impact is challenging yet several quantitative systematic reviews have reported a negative impact on children’s and YP’s mental health across demographic groups, research designs, and countries [14–19]. Depressive and anxiety symptoms were reported to have increased in YP compared to pre-pandemic [20–22] and mental health worsened in those with existing physical health problems [19]. This impact was pronounced early in the pandemic, but some evidence suggests this impact continued throughout Covid-19 [19]. In contrast, many adolescents and YP demonstrated resilience to the pandemic in the long-term [23], particularly following reduced lockdown measures (e.g., returning to school) [24]. Overall, quantitative study evidence is mixed [25] and needs further exploration.

Qualitative studies have explored European YP’s mental health and wellbeing in the context of the pandemic, but this evidence has not yet been synthesised using a co-produced approach. For a better understanding of the mental health and wellbeing impact from YP’s perspectives [26], a systematic review is warranted to build an informed and sensitive recovery response. Moreover, it is equally important to work with YP with lived experience of mental health difficulties to ensure the synthesis is relevant to YP, to meaningfully inform policy and practice. Unfortunately, this partnership has been relatively scarce during the pandemic, and no published reviews focusing on the impact of Covid-19, whether quantitative, qualitative or mixed methods, have been co-produced with YP. Our review aim was therefore to work with YP with lived experience of mental health difficulties to produce a qualitative systematic review examining the perceived impact of the Covid-19 pandemic on YP’s mental health and wellbeing. We asked:

1. What do YP identify as challenges facing them because of the Covid-19 pandemic?
2. How have YP been coping during the Covid-19 pandemic?
3. What is the perceived impact of the Covid-19 pandemic on YP’s mental health and wellbeing in the short- and long-term?

## Methods

### Managing the review, search strategy and selection criteria

**Fig 1:**
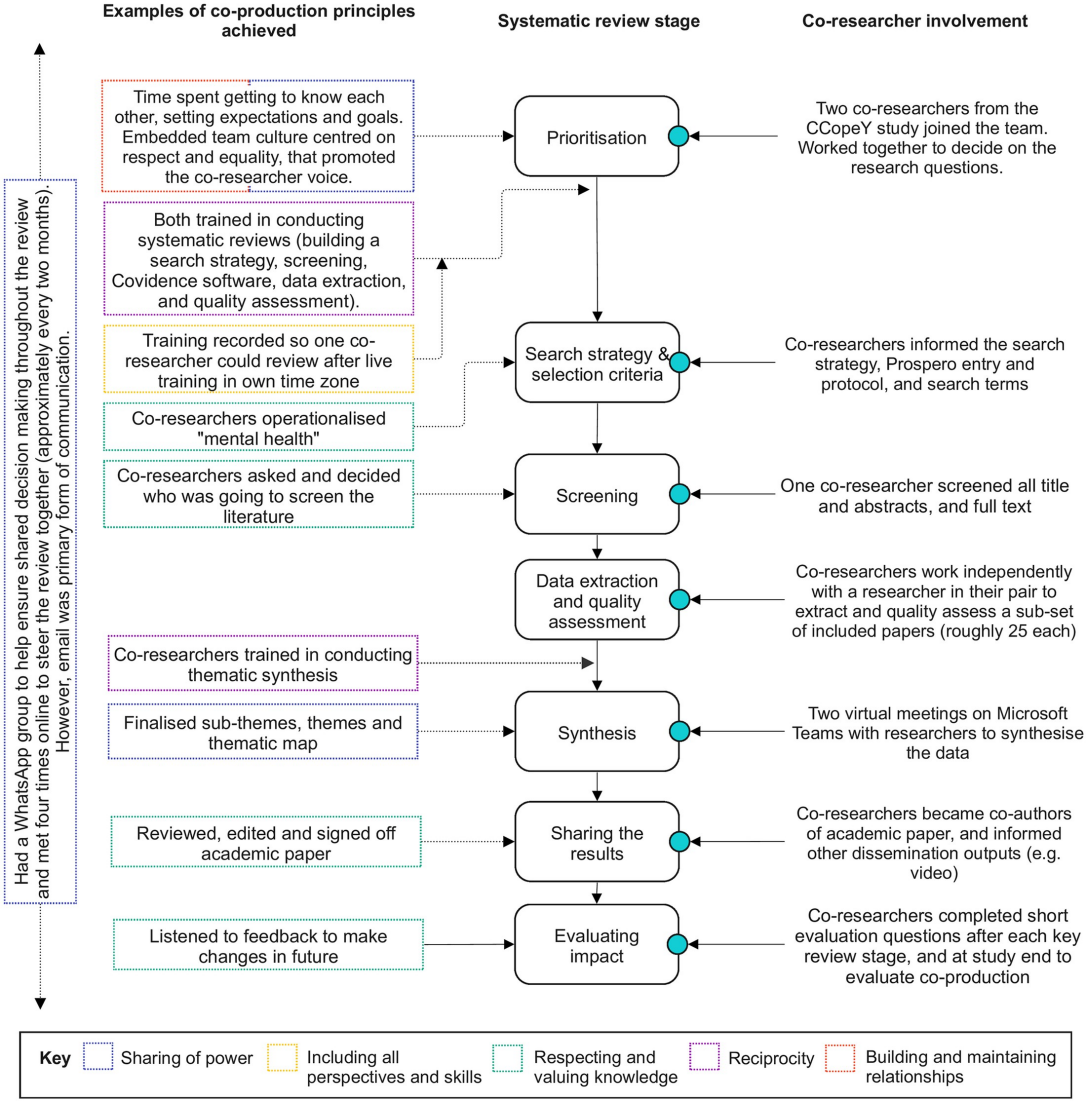
Co-production in our systematic review across all review stages.

We were guided by the Preferred Reporting Items for Systematic reviews and Meta-Analyses (PRISMA) statement and checklist [27] (S1 Checklist) and used Covidence to manage our review [28]. Two co-researchers aged 18 and 27 (CC, EC) with lived experience of mental health difficulties were involved throughout all review stages from conceptualisation to dissemination. Involvement is reported and incorporated throughout the main review sections. Both were supported throughout and paid in accordance with NIHR INVOLVE guidance [29]. NIHR principles of co-production (Fig 1) and the Guidance for Reporting Involvement of Patients and Public (GRIPP2) short-form were used (S2 Checklist) [30]. This review was registered with PROSPERO (ref: CRD42021251578).

### Search strategy

The review search strategy was developed using the SPIDER framework [31]. This was chosen to provide a systematic strategy for searching qualitative research and is adapted from the PICO tool often used for quantitative research. This is broken down for our review specifically as follows:

**Box 1. SPIDER strategy.**

Sample: Young people aged 10–24.

Phenomenon of Interest: Mental health and wellbeing during Covid-19.

Design: Qualitative and mixed methods research and specific qualitative methods i.e., semi-structured interviews.

Evaluation: N/A.

Research type: N/A.

An initial search strategy was tested within MEDLINE, developed, and improved across two team meetings and discussions with an institutional librarian. Our final strategy was sensitive against our initial strategy and produced similar hits. We searched with Medical Subject Headings (MeSH) and applied the same search string adapted to each database. Final search terms and Boolean operators aligned with the SPIDER tool and were across four facets: sample, phenomenon of interest: mental health and wellbeing, phenomenon of interest: Covid-19, and design: qualitative and mixed methods (S1 Text). We screened reference lists from each included paper to identify additional papers for inclusion.

MEDLINE, PsycInfo (Ovid), Embase, Web of Science, MedRxiv and OSF preprints were searched by one researcher (LR) from 1^st^ January 2020 to 15^th^ November 2022. Grey literature was screened in Google on three separate dates (11^th^ August 2021, 22^nd^ September 2021 and 12^th^ December 2022), until there were 20 consecutive irrelevant results. We requested additional literature through Twitter, existing contacts, relevant charities’ websites, and international mental health registers (e.g., Covid-MINDS) between 16^th^ and 23^rd^ September 2021 and again on 9^th^ December 2022 (S1 Text). Following deduplication, two reviewers (LR, EC) independently screened titles and abstracts and then full-text. Consensus was achieved. Data was independently extracted across three researcher pairs (e.g., researcher and co-researcher together where possible; LR and EC, OD and CC, and EA and SS), to support reliability and reduce bias [32]. OD did this alone for results from the second search on 15^th^ November 2022 with LD to resolve disagreement. A seventh researcher (LD) resolved disagreements across all review stages.

European studies were included if qualitative findings on YP’s experiences, views or perspectives of Covid-19 and related disruptions to their mental health and wellbeing could be extracted (e.g., non-numerical descriptive data). YP were aged 10–24 but studies were included if the sample covered the lower or upper range (e.g., aged 16–25). We focused on European countries to bring an international context for application to practice and policy changes. We conceptualised mental health and wellbeing from the WHO definition [33], and through discussion with co-researchers to capture various facets including thoughts, feelings, and behaviours. Outcomes relating to mental health and wellbeing of YP therefore included: mood, emotions, coping, anxiety, and suicidality. Peer- and non-peer- reviewed outputs (e.g., pre-prints, reports) were considered. We excluded quantitative studies that did not include qualitative data (e.g., experiments), non-empirical studies (e.g., editorials, protocols, and commentaries), reviews, case studies, conference abstracts and proceedings, book chapters, and studies where qualitative findings or views of YP could not be extracted (e.g., combined thematically with views of parents/teachers). Non-English language papers were also excluded, however, only three papers were excluded for this reason.

### Data analysis

The primary outcome was the impact on YP’s mental health and wellbeing. Adapted thematic synthesis was used to analyse and synthesise information in four key stages [34]. First, reported themes and sub-themes from the included papers were extracted independently by researcher and co-researcher pairs where possible (LR and EC; EA and SS; OD and CC) and transferred into Trello (online project management tool). Second, where manuscripts lacked clear themes, researcher pairs subjected them to line-by-line coding and described and summarised relevant text. Codes were then grouped into themes or deleted if identified as a repeat by two researchers (LD, LR). Third, across three 2-hour virtual analysis meetings, each pair blended, grouped, or deleted codes independently across each research question, resulting in sub-themes. We discussed discrepancies or concerns, refining sub-themes and filtering out replicates or irrelevant information. Sub-themes were transferred into Miro (online visual collaboration platform) and colour-coded by review question. Two researchers (LD, LR) then created an initial thematic map which was refined through an iterative process (three subsequent rounds) with the wider team (EC, EA, OD, SS). Sub-themes were linked where possible, and overarching themes were developed to reflect similarities among sub-themes. To maximise trustworthiness, we made the data extraction process transparent, maintained an analysis audit trail, and engaged in team discussions to challenge formative themes.

Included peer reviewed studies were assessed for quality across researcher pairs (LR and EC; EA and SS; OD and CC) using the Mixed Methods Appraisal Tool (MMAT) [35]. Any disagreements were resolved in discussion with an independent researcher (LD). Individual studies were classified as meeting 25%, 50%, 75, or 100% of MMAT criteria [36]. One independent researcher (LR) used the CERQual [37] approach to assess confidence in (i) methodological limitations, (ii) coherence of results, (iii) adequacy and sufficiency of data and (iv) relevance. Two researchers (OD, LD) validated the findings from the confidence assessment. A classification of low, moderate, or high confidence was assigned to each theme according to the CERQual confidence assessment.

### Positionality

Qualitative research embraces the influence of research subjectivity and that is essential to ensure trustworthiness in findings. We acknowledge the positionality, experiences and background of the research team that conducted the data extraction, synthesis, and interpretation of findings in this review (CB, CC, EA, EC, LD, LR, OD, SS) as part of reflexivity. The team represents varied ages, ethnicities, universities, country origins (e.g., UK, Portugal, and wider Europe) and experiences of mental health, wellbeing and Covid-19 both from a personal, and carer position. The two co-researchers have disclosed specific lived experience of mental health difficulties and knowledge of their peer’s experience of mental health, wellbeing, education and Covid-19 living in the UK (CC, EC). Their involvement was crucial to understanding the young person perspective. Our collective diversity allowed for individual and group reflections on our potential biases towards the analysis.

### Role of the funding source

We received funding from the NIHR Imperial Patient Safety Translational Research Centre (PSTRC-2016-004) for co-production costs. The funder had no role in study design, data collection, data analysis, data interpretation, or writing the report.

## Results

### Description of studies

**Fig 2:**
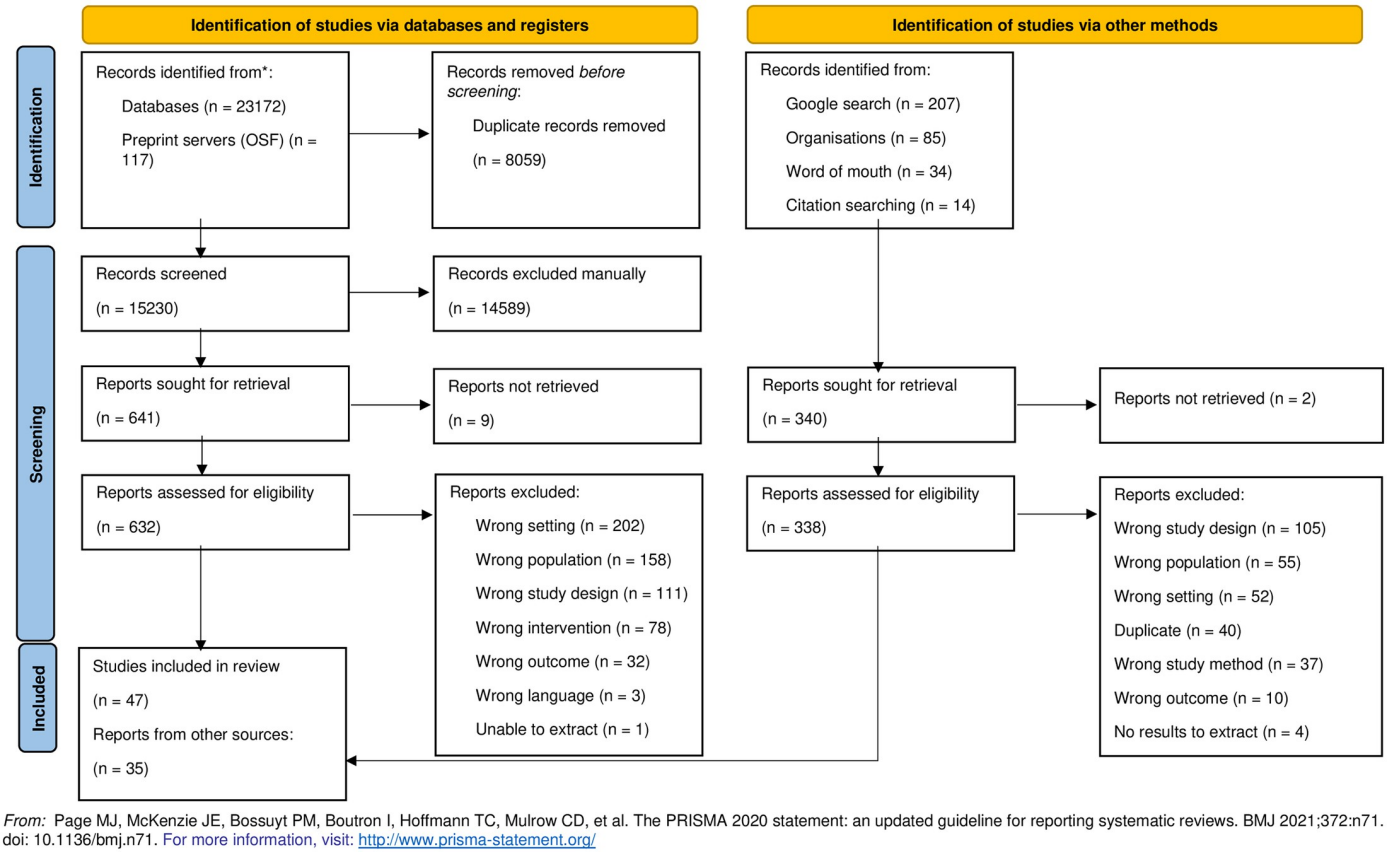
Flowchart of studies.

From 23,629 hits, we included 82 publications reporting on 77 unique studies (Fig 2, S1 Table) [38–115]. Fifty-one (66%) were peer-reviewed journal articles. Study designs were either qualitative (n = 47; 61%) or mixed methods (n = 30; 39%) The methodology used to generate qualitative data varied and included open-response survey questions (n = 28; 36%), semi-structured interviews (n = 22; 29%), focus group discussions (n = 13; 17%), in-depth interviews (n = 5; 6%), multi-genre narratives e.g. poetry or photography (n = 3; 4%), narrative diaries (n = 3; 4%), narrative accounts (n = 2; 3%), participatory drawings (n = 2; 3%), consultations (n = 2; 3%), ethnography (n = 1; 1%), open-ended structured interviews (n = 1; 1%), insights (n = 1; 1%), and semi-structured interviews with peers (n = 1; 1%). Over half used thematic analysis (n = 47; 61%), two-thirds did not describe their conceptual or philosophical framework (n = 31; 66%), and less than a quarter of studies reported on patient and public involvement; only one study reported impact of involvement.

**Fig 3:**
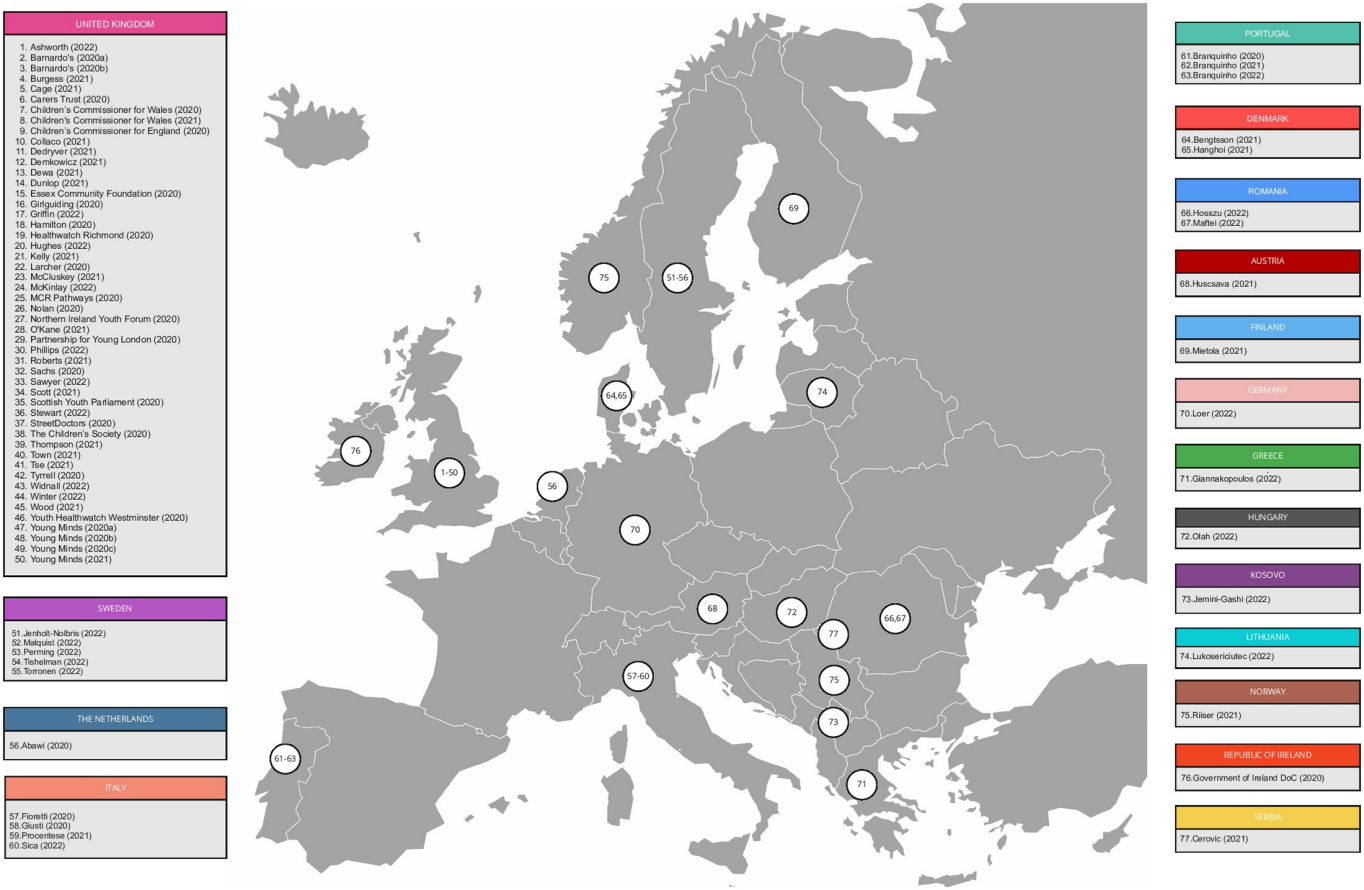
Map of studies in Europe. Reprinted from Wikipedia Commons under a CC BY license, with permission from Wikimedia Commons, the free media repository, original copyright 2011.

**Fig 4:**
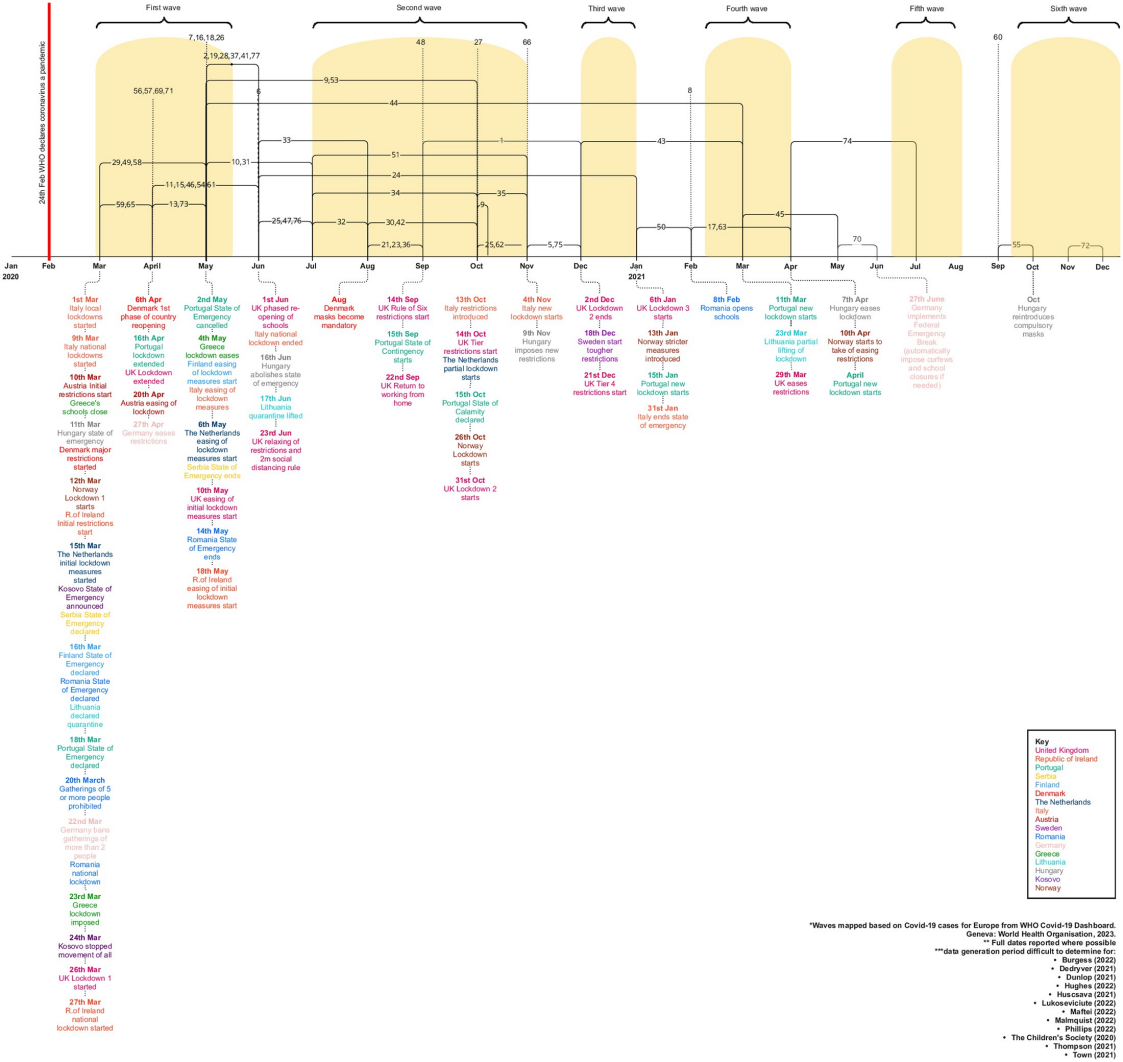
Qualitative data generation periods against key Covid-19 milestones.

Most were UK studies (n = 50; 65%) and remaining countries included Sweden (n = 5; 6%), Portugal (n = 3; 4%), Italy (n = 4; 5%), Denmark (n = 2; 3%), Romania (n = 2; 2%), Serbia (n = 1; 1%), Finland (n = 1; 1%), Ireland (n = 1; 1%), Lithuania (n = 1; 1%), Norway (n = 1; 1%), Austria (n = 1; 1%), Germany (n = 1; 1%), Greece (n = 1; 1%), Hungary (n = 1; 1%), Kosovo (n = 1, 1%) and The Netherlands (n = 1; 1%) (Fig 3). There were 79,491 participants across included studies; eleven studies had between 1,000–8,000 respondents, and two had over 19,000. Reporting of demographic information varied; 75% of studies reported gender (n = 58), of which most predominantly included female participants. It was not possible to determine total proportion ethnicity or sexual orientation as only 36% and 6%, reported a breakdown (n = 36 and n = 5, respectively). Participants were aged 4 to 30 years, but few studies reported mean participant age. Data generation dates varied, but most (n = 33; 43%) explored the first Covid-19 wave (first 100 days of the pandemic), which began during the first lockdown in most countries (Fig 4).

### Main findings

**Fig 5:**
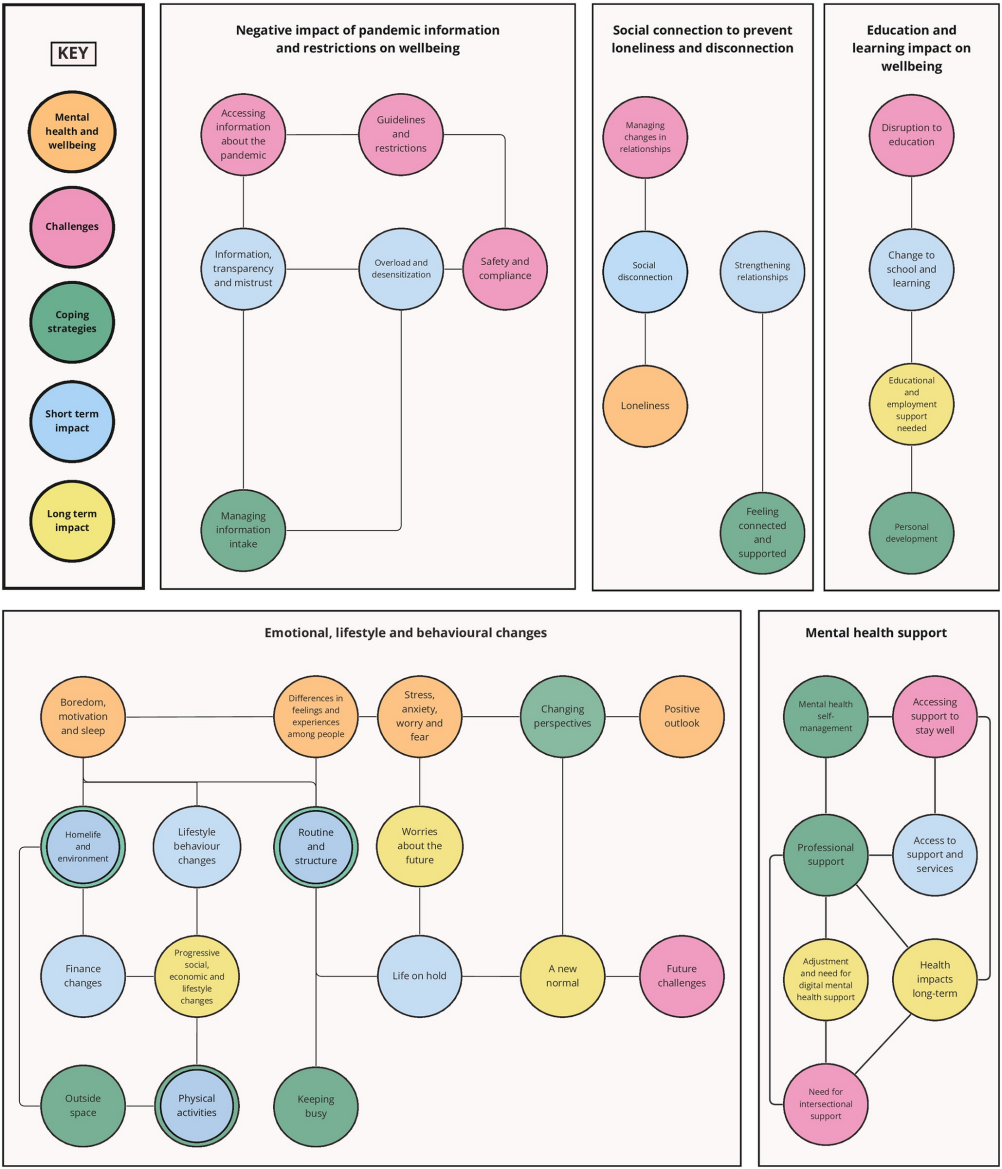
Co-produced thematic map.

Thematic analysis resulted in five main themes, each with subthemes relating to multiple review questions: negative impact of pandemic information and restrictions on wellbeing; education and learning impact on wellbeing; social connection to prevent loneliness and disconnection; emotional, lifestyle and behavioural changes; and mental health support (Fig 5).

#### Negative impact of pandemic information and restrictions on wellbeing

Regular news updates about pandemic deaths and regulations were reported as challenging by YP, particularly the constant access (e.g., news notifications on mobile phone) and scheduled updates (e.g., television government updates). Consequently, some reported managing this by avoiding social media completely for periods of time [53]. Similarly, constant changes in guidelines and restrictions such as introducing different modes of social distancing (e.g., the ‘rule of six’ in the UK) and national/localised lockdowns created uncertainty and confusion for some YP regarding what was expected of them. Indeed, some reported being compliant with the rules while feeling resentful of peers they felt were not [52, 53, 89, 93]. For example, young people aged 16–18 years in Norway and aged 13–17 in the UK felt not abiding by rules was selfish, especially when some reported sacrifices such as being alone in hospital [89].

“*At the beginning I felt like only some of us took the responsibility*, *so I had to think about it for everyone*, *like ok*, *we’re too many*, *so I can’t join*, *but all the others are going*, *so it was kind of*… *everyone else was joining*, *but I couldn’t because then we were too many*.”

(Female) [89]

“*I have had multiple fallings out with one of my best friends*. *Because she just doesn’t understand*, *well*, *no*, *she understands why*, *obviously*, *but she just won’t stop going out*. *And I’m like*, *what are you missing out on*, *you’ll see everyone at school in a couple of weeks*, *I don’t know*, *that got me really frustrated*, *why can’t people just understand*, *sacrifice these next few months*, *stay inside*, *and you’ll see everyone back to normal*.”

(Female, aged 16) [93]

#### Social connection to prevent loneliness and disconnection

Most YP Europe-wide reported having difficulty in managing changes in relationships, including not seeing others face-to-face, experiencing isolation, and poor-quality connection with others online. For example, YP, largely from the UK, felt connecting online was just not the same as in-person, and some had rarely used technology in this way prior to lockdown so found this new [79, 114]. For one young person aged 11 in the Netherlands, the perceived vulnerability of catching Covid-19 for their parents meant they were not permitted to see friends even when rules allowed it [39]. This restriction to seeing friends and disconnect from others created a feeling of social isolation [43, 45, 57, 64, 67, 78, 81, 90, 93, 96, 108, 112, 115], loneliness [43, 45, 53, 57, 61, 64, 81, 90, 93, 95, 96, 98, 112, 114, 115], and being forgotten [43, 64]. For example, some older YP aged 18–29 years in Denmark reported feeling forgotten by healthy peers, and therefore felt alone with their negative thoughts [64]. Some felt loneliest during the early stages of each lockdown. However, one of the few studies that captured perspectives past Wave 2 (Fig 4) found that these feelings of loneliness and social isolation continued long-term [115].

"*I have felt incredibly lonely despite having a great support system and being in the same household as one of my best friends*, *my sister*."

(Unknown) [52]

“*Before lockdown*, *I never used video calls or phoning people anyway*. *I spoke to people through messaging a lot*, *but I never used Zoom*. *I never heard of it to be honest*. *So*, *when I was suddenly forced to do that*, *it was just too much*, *so I’ve stopped talking to people*.”

(Male, aged 13–17) [79]

In contrast, others reported feeling *more* connected to others, and supported, even online. For example, YP (age not reported) from Denmark used gaming as a way of maintaining social relationships beyond their households [44]. For others in the UK, connecting online through video calls and social media platforms or face-to-face in line with pandemic rules (e.g., socially distanced) helped them remain connected with others [53].

“*I think that definitely yes*, *social media has helped a lot*. *At the beginning we were using House Party and there have been others since*. *It’s nice to see YP use social media to keep in touch and be innovative*. *It’s been good to see younger people teach older people what to use also*.”

(Young person who identifies as minority ethnic) [38]

Similarly, for other YP, the Covid-19 pandemic significantly improved relationships with family, friends and partners [40, 45, 47, 53] which led to feeling socially connected [44]. Indeed, the quality of the connection with others was reported to be more important for YP in the UK than the physical proximity of the friendship group [53].

“*I guess I’ve come to see how lucky I am to have all these friends*”

(Male) [40]

“*I won’t take for granted as much*, *like just standing outside talking to my neighbour*, *phone calls with my [relative]*. *Just the little things like that*. *Because obviously it’s so easy in normal life just to brush them aside and say*, *‘oh*, *I’ll ring my [relative] later*.*’ So now I take the time to ring my [relative] and talk to her*.”

(Female, aged 13–17) [79]

#### Education and learning impact on wellbeing

Changes to school routine, delivery, and learning was reported to be a significant short-term impact on YP during the pandemic, across all studies and ages [38–40, 42–45, 47, 48, 52–58, 60, 61, 63, 64, 67, 71, 72, 74, 78–81, 84, 88, 90, 91, 93, 95, 96, 98, 102–106, 108, 112–115]. This disruption to education and learning was therefore a significant challenge for most, regardless of the pandemic milestone, and was a strong thread across most studies (Fig 4). Some YP expressed their frustration with relying on “predicted grades” rather than recognising the increased effort and difficulties they experienced while learning online.

“*Not doing exams*, *I was gutted*. *I can’t prove myself and I’ve being given a letter that they think I could get*… *they didn’t see how hard I was working*…*they don’t see how hard we work outside of school*. *And even if*, *say*, *someone got a D but they pushed so hard trying to get an A and they were getting there*, *they were just trying so hard*. *They don’t see that*, *they don’t see everything we do in school*. *It’s just annoying that we can’t prove ourselves*.”

(Female, aged 13–17) [79]

“*Often*, *I think that what if there was no corona*, *what would I have experienced*? *We were barely halfway through our first year in upper secondary when*… *I just feel that I don’t grow as a person*, *I’m stuck in the first year and then time goes by and suddenly upper secondary school will be over*.”

(Male) [89]

However, one UK study reported that taking part in school online, rather than in person, was beneficial to the mental health of YP with pre-existing mental illness [106].

“*Not going to school has helped me in many ways; it gave me time to recover from being in poor mental health and gave me a break from all the hassle of school days*.”

(Identified as trans, aged 13) [106]

Long-term, these educational changes were perceived to have an impact on exam results, readiness for future educational milestones, and general performance. As such, some YP highlighted the need for additional educational support to catch up and help them prepare for the next steps in their lives. In Serbia, participants (aged 12–18) highlighted the need to support learning during lockdown but in a more creative and less demanding environment [71]. In contrast to traditional educational or employment concerns, a core sub-theme was coping with these changes through personal development and social exploration. Many YP reported using lockdown as an opportunity to escape normal life pressures, explore themselves through new activities (e.g., new hobbies), and were able to continue these activities after restrictions had ended. Indeed, 14–20-year-olds from Italy reported having more time for self-discovery and personal growth [56].

“*I like being able to do things like courses and workouts I didn’t previously feel I had time for*”

(Unknown) [52]

#### Emotional, lifestyle, and behavioural changes

Most studies reported that the Covid-19 pandemic had a negative impact on YP’s mental health and wellbeing. This included perceived low mood, stress, anxiety, worry and fear of the unknown, as well as boredom and being demotivated without routine. Many YP across countries described feeling these emotions in the present moment but also in relation to their future. However, almost all studies reported fluctuating emotions and changes in lifestyle behaviours as the pandemic progressed, which painted a complex picture [38–40, 42–45, 47, 48, 52–58, 60, 61, 63, 64, 67, 71, 72, 74, 78–81, 84, 88–91, 93, 95, 96, 98, 102–106, 108, 112–115].

“*I have been finding it hard to deal with the uncertainty of it all*. *I also struggle with lack of structure and routine*.”

(Female, aged 15) [63]

“*I feel sad most of the time now and some days I can’t even get out of my bed because I just think there is nothing for me to do anyway*. *Then I feel anxious about schoolwork and that I’m behind*.”

(Unknown) [59]

These emotions were made more complex because their expression differed between and within studies. For example, some YP across the UK and Italy reported amplified emotions compared to normal, such as intense anger, sadness, and grief [42, 53, 56, 57, 74, 79]. In contrast, some YP, such as psychiatric outpatients living in Austria [67], instead felt their emotions fluctuated regularly, often because of uncertainty that came with changes in guidelines and potential future challenges [39, 79, 90, 108].

“*I alternate between anxiety so bad I shake and cry and can’t concentrate on anything and then depression so bad that I can’t get out of bed*. *I’m also so scared of being infected (and then infecting others) that I haven’t left my house in nearly 100 days*.”

(Unknown) [115]

"*I’ve already got a history of mental health issues*, *being shoved into a house with none of my friends and any sense of normality shredded has certainly not helped*."

(Unknown) [39]

“*It has been a roller coaster of ups and down since the very day schools closed*, *some days have been extremely low*, *there were weeks where I cried every day and found just getting up challenging*.”

(Female, aged 18) [61]

Other studies reported on mental health differences across groups, such as minoritised groups, [38, 42, 43, 48, 53, 54, 70, 74, 77, 80, 81, 90, 91, 98, 102, 103] those with experience of a specific condition [39, 49, 50, 104] or mental health difficulties prior to the pandemic [59, 60, 67, 81, 104, 112–115]. All UK studies exploring experiences of LGBTQ+ YP aged 12–25 [42, 43, 54, 102], reported maladaptive coping mechanisms such as self-harm [54, 102], (although one study specified experience of self-harm as a recruitment criterion), and alcohol or drugs [43, 54]. In Sweden, young people who identified as queer also reported suicidal thoughts, or fear of their friends dying by suicide [77].

“*For a little period*, *I self-harmed*… *I was kind of looking for an outlet anywhere I could find at that moment*. *And*, *I’d*, *I’d never feel better afterwards*, *I’d feel worse*

(Unknown) [102]

A minority of studies reported YP having a positive outlook and trying to stay hopeful. This was reflected in how some YP reported that they were coping with uncertainty, embracing the lockdown and accepting “a new normal” that may remain long after lockdown measures end.

“*In a very positive way*, *the time I have is perfect for investing in me*, *in my development*, *organizing the life that was previously on the run and taking the opportunity to be with those who share the same roof*.”

(Female, aged 19) [45]

“*Positive reappraisal was something I did quite a lot right at the start*… *trying to find like things I can do that I couldn’t do before so I can see the good side of everything*… *I feel like this is a good time for kind of re-examining myself and really trying to focus on what I want to accomplish*”

(Male, age unknown) [53]

Nevertheless, many studies reported that YP felt their lives were on hold during the pandemic, and that they were missing out on life opportunities. Short-term impacts on routine and structure were experienced by many across studies and countries. For example, some UK-based YP who had previously requested mental health support expressed that the cessation of activities, such as exercise and dance classes, worsened their anxiety [113]. However, many kept busy, and maintained their usual routine as much as possible (e.g., get up at set time, brush teeth, etc.) or adapted to a new routine (e.g., “eat, sleep, revise, repeat”) [105] that involved helpful coping strategies. Other reported short-term impacts were “financial”, “homelife and environment” and “lifestyle behaviours”. For instance, unemployment or being furloughed meant some YP or their families were on reduced salaries, leading to worry and anxiety about how they would afford bills [74, 103].

“*Money is always a worry because of the huge delay in any payments and only getting 80% [of their salary due to being furloughed]*, *things were tight before*, *they are worse now*. *We use more food and utilities but there is no help for this*.”

(Young adult carer, aged 19) [103]

“*It has been a real struggle to even find 3 meals in a day*… *let alone how the electric will be topped up*. *In terms of my current employment—it is a casual contract and therefore we do not have much coming in with this either*. *It’s a real struggle however we stay grateful that we are not homeless and have each other*”

(Unknown) [74]

#### Mental health support

Most YP mentioned practicing self-care or using self-management techniques to manage their mental health and wellbeing. Some techniques that were common between countries or groups included distraction and keeping busy [53, 54, 81, 88, 93, 95, 112], physical exercise [40, 43, 45, 53, 81, 96, 112], spending time with pets [38, 43, 47, 53, 81], cooking [40, 45, 56, 81, 108], online gaming with friends [44, 45], listening to music [40, 45, 53, 64, 81], and reading [40, 45, 56, 64].

“*I read*, *studied*, *I’ve cooked various stuff*, *experimented*, *relaxed taking time for myself*, *watched TV series*, *movies*, *played chess*. *Everything that made me feel good*. *I felt accepted by myself*, *because I had time to think about myself much more and to reflect*, *making me feel like a better and acceptable person*”

(Male, aged 15) [56]

“*My dog has gave me purpose to get up*, *dressed and go for a walk*”

(Young person with previous experience of the youth justice system) [81]

However, many simultaneously reported poorer diets and sleep. Consequently, in the long term, some YP reported the desire for the government to prioritise their mental health and wellbeing.

“*What are you going to do to improve the treatment of children with mental health conditions in Northern Ireland when the CAMHS system can’t even afford to take everyone who needs their help when they need it*?”

(Aged 15–17) [57]

The need for mental health support during the pandemic was challenging for YP, both for those with pre-existing mental health difficulties and those who developed difficulties during the pandemic. For example, these groups reported a lack of informal support (e.g., non-medical sources), stigma to seeking help, appointment cancellations, and general barriers to accessing support. Barriers to accessing support included not being able to have face-to-face appointments [43, 61], long wait-lists [53, 108, 115], and finding it difficult to establish trust or rapport with mental health professionals online [42, 64]. In contrast, some YP reported experiencing good professional support including receiving wellbeing catch-ups at school or tele-mental health support [53, 64, 67, 79, 90, 98, 112, 113].

Others reported that having peer and family support was beneficial in the absence of professional support [38, 45, 48, 56, 61, 90, 91, 98, 105, 108], whereas others did not have either and were struggling alone [38, 42, 53, 58, 61, 64, 67, 79, 84, 90, 112–114]. There was a reported need for intersectional support throughout the pandemic because of inequality in accessing and receiving support. Therefore, the continual need for receiving face-to-face professional mental health support, as well as adjustment to more accessible digital support for all, was perceived to be important for YP in the short- and long-term.

“*Staying at home brings me moments of nervousness and I’m easily irritable*. *I often have panic attacks*, *precisely because staying at home for so long is not good for me*. *One feels alone*, *like in a cage and suffocated feelings give rise to nervousness that causes tension*”

(Female, aged 16) [56]

“*And you may not think that it means much*, *like a lot*, *but it does*. *For somebody*, *especially in lockdown when you think that you’re all alone*, *and to have a message saying*, *‘how are you doing’ or ‘just wanted to check up on you’ or something*, *it gives you a boost of confidence to know that there are people out there that are willing to help you if you are struggling*.”

(Unknown) [90]

#### Quality appraisal of included studies

Fifty unique publications were subjected to quality assessment using the MMAT tool [39, 40, 44–54, 56, 59, 61, 62, 64–73, 75–79, 82, 83, 85–87, 89, 90, 92–94, 97, 99–102, 104, 109, 110] (S2 Table). Forty-three studies met 100% of MMAT criteria; [39, 40, 44, 48–54, 56, 59, 62, 64–70, 72, 73, 75–79, 82, 83, 85, 87, 89, 90, 92–94, 97, 99–102, 109, 110] four met 75% [46, 47, 61, 86]; one met 50% [104] and two studies met 25% [45, 71]. The primary shortfall was inadequate reporting of mixed methods studies. Study findings’ confidence ranged from low to moderate (S3 Table). Study findings mainly represented subgroups of the European youth population. Additionally, some studies’ methods or links between themes and raw data were unclear.

## Discussion

To our knowledge, this is the first qualitative systematic review to explore the perceived impact of the Covid-19 pandemic on YP’s mental health and wellbeing Europe-wide. From 77 unique studies across 17 European countries, there were five themes that interconnected challenges, coping strategies, and perceived impact of the Covid-19 pandemic on mental health and wellbeing: negative impact of pandemic information and restrictions on wellbeing; social connection to prevent loneliness and disconnection; education and learning impact on wellbeing; emotional, lifestyle and behavioural changes; and mental health support. YP perceived the pandemic to have worsened their mental health and wellbeing, particularly across the initial stages, despite some having positive outlooks. This was in line with most quantitative evidence synthesis [17, 116], but one quantitative review indicated mental health had not significantly worsened since Covid-19 [23]. In contrast, our review showed the nuances of this complex picture. Perceived stress and anxiety related to the pandemic continued throughout Waves 1, 2 and 3 across Europe; and many YP also worried about their future. This contrasts with some quantitative evidence that suggests YP were resilient, and their wellbeing improved once they returned to school [24]. Similar short-term challenges that impacted wellbeing across the studies included education disruption (e.g., exam cancellation, remote teaching), information overload, managing changes to routine, guidelines, and restrictions, and limited mental health and wellbeing support. However, there were also complex considerations that extended into the long-term, with YP describing having missed opportunities and key social milestones that they will never recover. Indeed, the significance of education disruption on future wellbeing was reported by participants as largely overlooked by government policies.

Whilst other (predominantly short-term) impacts of the pandemic varied, most studies reported YP experiencing impacts on lifestyle behaviours (e.g., sleep), disconnection with others, and loneliness. This aligns with previous quantitative reviews that reported high prevalence of loneliness among YP during the pandemic compared to pre-pandemic [19, 117]. Therefore, strategies to tackle loneliness among YP are vital across Europe [118]. Improving social connection can protect against loneliness and deteriorating mental health [119, 120]. The strong emphasis on good quality connections with others in our review further strengthens the importance of maintaining relationships with others through peer, family and professional support [121] during times of crisis such as climate change, and therefore should be a possible tractable intervention target. The continued ubiquitous use of digital technologies since the pandemic and recognised importance of digital social connection as an “active ingredient” to improving YP’s mental health [119], means there may also be a need to adapt and apply current evidence-based interventions to digital contexts.

YP reported the need for better access to mental health support and services, and professional support. Indeed, barriers to accessing mental health care include systemic and structural factors (e.g., lack of availability, long-wait lists, financial constraints), social factors (e.g., perceived stigma), individual factors (e.g., limited mental health knowledge) and therapeutic relationships with professionals (e.g., inability to have face-to-face appointments, establish rapport). These barriers echo those identified before the pandemic [122], indicating continuity of the complex array of factors preventing YP from accessing professional support. However, pre-existing inequalities to accessing mental health care have worsened since the pandemic [123]. We recognise that barriers intersect across multiple axes of inequality such as ethnicity, gender identity, sexual orientation, and rurality, and therefore, intersectional support should be a key future priority.

### Strengths and limitations

To our knowledge, no other Covid-19 focused review has been co-produced with YP with experience of mental health difficulties throughout all systematic review stages. This is crucial to achieve moral, epistemological, and consequentialist arguments of involving people with lived experience in research [124]. Meaningfully involving co-researchers has resulted in robust and sensitive mental health operationalisation, triangulation of thematic analysis, and a wide coverage of publications, including grey literature from charities that may have been otherwise overlooked. We have reported the study according to the GRIPP2 checklist [30] to emphasise our commitment to co-production and meaningful involvement. More broadly, our review was registered on PROSPERO, and systematically conducted using accepted methodology, including double screening to ensure enhanced rigour and replicability. Our mapping of publications by country and across pandemic milestones means our review can easily operate as an accessible index resource for policy-forming organisations and clinicians, and evidence the gaps in data generation across Europe.

Our review also has limitations. Whilst we covered publications from across the initial three waves of the pandemic, more work is needed to explore qualitative literature examining the long-term impacts on YP’s mental health and wellbeing as we move beyond pandemic recovery into a world where we are expected to live with Covid-19. Our included studies mainly focused on the UK perspective, and perspectives in other European countries may have differed substantially; there was a paucity of qualitative literature focusing on specialised groups across Europe. This largely explained the low degree of confidence of some sub-themes using the CERQual approach. Therefore, synthesis was difficult and YP’s voices minoritised by demographic status or condition were underrepresented, potentially biasing understanding of YP’s experiences. For instance, there were no studies that reported YP’s experiences in prison, being homeless, or with learning disabilities. We also did not compare “like for like” experiences across different factors (e.g., experience, diagnosis, context). Whilst this was not the aim of our review, we acknowledge this would be a useful exercise in the future to explore more complex nuances. Finally, we only worked with two co-researchers, and one was aged 27 at review submission. Whilst this was beyond our defined age range of 10–24 and we strove to match experience to study focus, this person started the CCopeY study [53] at aged 24 and had appropriate experience as a young person, with mental health difficulties and Covid-19. Despite our commitment to a co-produced approach, on reflection, we could have included a more diverse group of co-researchers, including younger people, men or those minoritised for different reasons, to strengthen the *including all perspectives and skills* co-production principle (Fig 1) [29].

### Research implications

There remains comparatively little qualitative data on YP’s experiences of the pandemic and the impacts on their mental health. YP’s perspectives from minoritised and marginalised groups, and those with specific physical health conditions are still lacking. Moreover, there were only two qualitative studies on YP’s experiences from low- or medium- income countries across Europe (Serbia [71] and Kosovo [68]). There is an urgent need for qualitative research to bridge these gaps, to tailor learning for changes in policy and practice for these groups. Although not the aim of our study, we found little patient and public involvement conducted across the 77 unique studies. This is in line with other quantitative systematic reviews exploring the pandemic and YP’s mental health [14–19]. Going forward there is an imperative need to work with people with lived experience of mental health difficulties on systematic reviews throughout all stages, to help improve the impact of synthesis, quality assurance and recommendations. In our qualitative review, we found only 77 studies and 50 were peer-reviewed. In contrast, to our knowledge, there have been 8 systematic reviews on the impact of Covid-19 on YP’s mental health and wellbeing that collectively found approximately 250 quantitative papers [14, 16–21, 25]. To achieve the same parity as quantitative research, we need to highlight the importance of qualitative research, and encourage funders to provide opportunities in this area.

### Clinical and policy implications

Our review informs clinicians, educators, and policymakers on European evidence of YP’s perspectives of their mental health and wellbeing during the Covid-19 pandemic. It highlights several key overarching areas that could help improve YP’s mental health and wellbeing across Europe during and following the pandemic or in similar crises. First, it is important that decisions regarding YP occur in partnership with YP themselves going forward. YP can make decisions about their own mental health and wellbeing, and healthcare professionals should use various methods to incorporate their views, such as co-production, advocacy and activism. Second, policymakers should acknowledge the impact the pandemic had on YP’s mental health and wellbeing. Those who experience inequalities should be prioritised to ensure that YP who have raised concerns are supported, particularly because YP minoritised by ethnicity [6], disability [7], or LGBTQ+ status [8] are already at particular risk of mental health difficulties. Furthermore, targeted evidence-based support should be accessible, and provided when and if necessary. Similarly, lifestyle behaviours such as good sleep should be promoted [125] and embedded from an early age, as part of universal prevention strategies. Finally, there are a range of considerations around educational disruption in both the short- and long-term, and curricula and examination procedures may require shifts in accountability due to lost learning. However, schools are not simply a place for academic progression–they can play a vital role in supporting YP’s social development and emotional wellbeing and provide a safe place for vulnerable children. Thus, there needs to be a continued focus on supporting YP to also ‘catch up’ on the vital holistic elements that schools provide. Subsequent recommendations are reported (Boxes 2 and 3) but we appreciate there are barriers to full implementation including resource limitations, structural inequalities and a more nuanced approach that consider individual and systemic factors. Drawing up a theory of change could be a useful next step to explore these recommendations.

**Box 2. Recommendations following the immediate aftermath of Covid-19.**

1. Give YP a seat at decision-making tables when considering their own mental health and wellbeing.
2. Provide accessible mental health support for which is tailored to individuals and groups where appropriate (e.g., minoritised and marginalised groups).
3. Do not assume that everyone has had a purely negative experience, and be aware that there are mixed, fluctuating and even positive aspects of the experience.
4. Prioritise universal promotion of self-care, wellbeing and lifestyle behaviours such as good sleep and physical health with the support of YP throughout the process.
5. Avoid pressure on academic ‘catch up’ and provide dedicated time in school to focus on wellbeing and social activities.

**Box 3. Recommendations following a future systemic crisis.**

1. Be considerate about the impact of news updates and how YP engage with this. This includes supporting YP in making sense of the information communicated to them on news and social media platforms and how it could affect them.
2. If social systems are disrupted, support YP in finding various ways to remain socially connected, including having an awareness that this may not be one size fits all.
3. Provide structure and routine for YP and ensure continued and uninterrupted access to physical and mental health and social care services.
4. Be aware of existing structural inequalities and how this intersects with emergent crises to ensure targeted support.
5. Support YP in navigating their coping responses. This should include an assessment of what is working for them and support them in finding alternatives where needed.
6. Be aware of those who do not want to connect digitally. Provide provisions to meet up face-to-face and ensure any technology can be available to everyone to support learning.
7. Have contingency plans for disruptions to education systems. If there are periods of online learning required, provide training and support for schools and educators in working flexibly and creatively with YP. Recognise that YP may struggle with self-regulation and managing a routine.
8. In instances of major disruption to education systems (particularly high stakes exams) teachers should have clear and regular consultation and communication with YP.

## Conclusion

Young people’s experiences map onto the quantitative evidence, indicating a largely negative experience of the pandemic and inequalities. Our review importantly explains the reasons behind these experiences, but these are complex, and formulate an interplay of quality social connections, appropriate mental health and educational support, and self-care. Many research gaps remain, such as the impact of the pandemic on minoritised and marginalised groups across numerous European countries. Notwithstanding another systemic crisis, such as climate change [126], widespread dissemination of recommendations highlighted in our review could help YP to not only survive another crisis, but to thrive.

## Supporting information

S1 Checklist PRISMA checklist. (DOCX)

S2 Checklist GRIPP2 short form. (DOCX)

S1 Text Search strategies. (DOCX)

S1 Table Study characteristics. (DOCX)

S2 Table MMAT. (DOCX)

S3 Table Confidence. (DOCX)
