# Supplementary material for: The impact of COVID-19 on young people’s mental health, wellbeing and routine from a European perspective: A co-produced qualitative systematic review
Source: PLoS One. 2024 Mar 20;19(3):e0299547. doi: 10.1371/journal.pone.0299547 (PMC10954119; doi:10.1371/journal.pone.0299547)
Supplement: S2 Checklist — (DOCX) [file pone.0299547.s002.docx]

**S2 Checklist. GRIPP2 short form.**

| **Section and topic** | **Item** | **Reported on page No** |
| --- | --- | --- |
| 1: Aim | Report the aim of PPI in the study | 7 |
| 2: Methods | Provide a clear description of the methods used for PPI in the study | 7 (Figure 1) |
| 3: Study results | Outcomes—Report the results of PPI in the study, including both positive and negative outcomes | 22 |
| 4: Discussion and conclusions | Outcomes—Comment on the extent to which PPI influenced the study overall. Describe positive and negative effects | 22, 23 |
| 5: Reflections/critical perspective | Comment critically on the study, reflecting on the things that went well and those that did not, so others can learn from this experience | 21 |
