# Supplementary material for: The impact of COVID-19 on young people’s mental health, wellbeing and routine from a European perspective: A co-produced qualitative systematic review
Source: PLoS One. 2024 Mar 20;19(3):e0299547. doi: 10.1371/journal.pone.0299547 (PMC10954119; doi:10.1371/journal.pone.0299547)
Supplement: S1 Text — (DOCX) [file pone.0299547.s003.docx]

**Supplementary material 3 Search strategies**

1. **Embase on 15/11/2022**

| **Search Domain** | **Term #** | **Search Term** | **Hit count (1960 total)** |
| --- | --- | --- | --- |
| Sample | 1 | adolescen*.mp | 1848559 |
|  | 2 | Young Adult/ | 412382 |
|  | 3 | Adolescent/ | 1738994 |
|  | 4 | boy*.mp | 241594 |
|  | 5 | Child/ | 2101060 |
|  | 6 | child*.mp | 3153309 |
|  | 7 | [CYP.mp](http://CYP.mp) | 21804 |
|  | 8 | girl*.mp | 231664 |
|  | 9 | juvenil*.mp | 171558 |
|  | 10 | minor*.mp | 437639 |
|  | 11 | Minors/ | 729 |
|  | 12 | p?ediatric*.mp | 703977 |
|  | 13 | pubescen*.mp | 3405 |
|  | 14 | prepubescen*.mp | 1280 |
|  | 15 | pupil*.mp | 54086 |
|  | 16 | school*.mp | 798281 |
|  | 17 | student*.mp | 547701 |
|  | 18 | [highschool.mp](http://highschool.mp/) | 184 |
|  | 19 | Students/ | 103974 |
|  | 20 | teen*.mp | 45664 |
|  | 21 | [young.mp](http://young.mp) | 1062457 |
|  | 22 | youth*.mp | 106864 |
|  | 23 | **AND** |  |
| Phenomenon of interest | 24 | COVID-19/ | 115 |
|  | 25 | COVID*.mp | 142063 |
|  | 26 | coronavirus.mp | 164068 |
|  | 27 | SARS-CoV-2/ | 14623 |
|  | 28 | SARS-CoV2.mp | 2470 |
|  | 29 | SARSCoV2.mp | 112 |
|  | 30 | SARSCoV-2.mp | 1008 |
|  | 31 | nCoV adj2 2019.mp | 1828 |
|  | 32 | 2019nCoV.mp | 18 |
|  | 33 | 2019-novel adj CoV.mp | 6 |
|  | 34 | nCoV adj 2019.mp |  |
|  | 35 | nCoV adj2 19.mp | 390 |
|  | 36 | severe adj acute adj respiratory adj syndrome adj coronavirus adj 2.mp |  |
|  | 37 | novel adj coronavirus.mp |  |
|  | 38 | novel adj corona adj virus.mp | 357 |
|  | 39 | corona adj virus adj disease adj 2019.mp | 708 |
|  | 40 | coronavirus adj disease adj 2019.mp |  |
|  | 41 | "novel coronavirus pneumonia".mp |  |
|  | 42 | "novel corona virus pneumonia".mp |  |
|  | 43 | quarantin*.mp | 14422 |
|  | 44 | self-isolat*.mp | 738 |
|  | 45 | lockdown.mp | 7640 |
|  | 46 | pandemic.mp | 116065 |
|  | 47 | lock adj down*.mp | 364 |
|  | 48 | dismiss* adj2 class*.mp | 36 |
|  | 49 | dismiss* adj2 school*.mp | 42 |
|  | 50 | school adj2 closure*.mp | 688 |
|  | 51 | social adj distanc*.mp | 12515 |
|  | 52 | shut-down*.mp | 2355 |
|  | 53 | shutdown*.mp | 3097 |
|  | 54 | stay* adj2 home.mp | 3240 |
|  | 55 | **AND** |  |
| Phenomenon of interest | 56 | (mental adj (health or ill-health or illness)).mp | 353813 |
|  | 57 | Mental Health/ | 159975 |
|  | 58 | agoraphobi*.mp | 7333 |
|  | 59 | wellbeing.mp | 90451 |
|  | 60 | well-being.mp | 125252 |
|  | 61 | psycho*.mp | 1820137 |
|  | 62 | Quality of Life/ | 513381 |
|  | 63 | "quality of life".mp | 654926 |
|  | 64 | QoL.mp | 81451 |
|  | 65 | emotion*.mp | 363995 |
|  | 66 | resilien*.mp | 48655 |
|  | 67 | challeng*.mp | 1180622 |
|  | 68 | stress*.mp | 1523342 |
|  | 69 | cope.mp | 49753 |
|  | 70 | coping.mp | 102529 |
|  | 71 | Anxiety/ | 237369 |
|  | 72 | anx*.mp | 448269 |
|  | 73 | Mental Disorders/ | 71577 |
|  | 74 | depress*.mp | 884447 |
|  | 75 | low adj mood.mp | 1533 |
|  | 76 | affective adj symptom*.mp | 3620 |
|  | 77 | dysthymi*.mp | 10719 |
|  | 78 | psychiatric.mp | 317907 |
|  | 79 | neurotic adj disorder*.mp | 1646 |
|  | 80 | panic.mp | 29597 |
|  | 81 | phobi*.mp | 31300 |
|  | 82 | dysphoric disorder*.mp | 1911 |
|  | 83 | affective adj disorder*.mp | 26584 |
|  | 84 | cyclothymic adj disorder*.mp | 155 |
|  | 85 | trauma*.mp | 598600 |
|  | 86 | trauma and stressor related disorders/ | 55562 |
|  | 87 | adjustment adj disorder*.mp | 5772 |
|  | 88 | mood*.mp | 158156 |
|  | 89 | PTSD.mp | 36604 |
|  | 90 | posttrauma*.mp | 95596 |
|  | 91 | post-trauma*.mp | 51220 |
|  | 92 | distress*.mp | 264997 |
|  | 93 | grief.mp | 15618 |
|  | 94 | grieving.mp | 2161 |
|  | 95 | self-injur*.mp | 7102 |
|  | 96 | self-harm*.mp | 9310 |
|  | 97 | attention adj deficit.mp | 71672 |
|  | 98 | attention deficit hyperactivity disorder/ | 65864 |
|  | 99 | ADHD.mp | 40060 |
|  | 100 | hyperactiv*.mp | 93959 |
|  | 101 | disruptive adj behavio?r*.mp | 6247 |
|  | 102 | SAD.mp | 15243 |
|  | 103 | fear*.mp | 140817 |
|  | 104 | frustrat*.mp | 26404 |
|  | 105 | happiness.mp | 13649 |
|  | 106 | happy.mp | 17537 |
|  | 107 | lonel*.mp | 13989 |
|  | 108 | self-esteem.mp | 36712 |
|  | 109 | self-mutilat*.mp | 2471 |
|  | 110 | Self-Injurious Behavior/ | 9531 |
|  | 111 | body adj image.mp | 27990 |
|  | 112 | suicid*.mp | 146086 |
|  | 113 | suicide/ | 63437 |
|  | 114 | self-killing*.mp | 84 |
|  | 115 | **AND** |  |
| Design | 116 | qualitative.mp | 370995 |
|  | 117 | participatory.mp | 20085 |
|  | 118 | interview*.mp | 544713 |
|  | 119 | focus adj group*.mp | 65591 |
|  | 120 | case adj stud*.mp | 208719 |
|  | 121 | mixed-method*.mp | 32806 |
|  | 122 | grounded adj theory.mp | 16328 |
|  | 123 | interpretative adj phenomenological adj analysis.mp | 2402 |
|  | 124 | semi-structured.mp | 82199 |
|  | 125 | IPA.mp | 10183 |
|  | 126 | unstructured.mp | 12816 |
|  | 127 | informal.mp | 27863 |
|  | 128 | in-depth.mp | 96714 |
|  | 129 | indepth.mp | 1919 |
|  | 130 | face-to-face.mp | 43243 |
|  | 131 | structured.mp | 262117 |
|  | 132 | guide.mp | 293795 |
|  | 133 | ethnograph*.mp | 14134 |
|  | 134 | fieldwork.mp | 4166 |
|  | 135 | field adj work.mp | 3244 |
|  | 136 | key adj informant*.mp | 9873 |
|  | 137 | narrative.mp | 50757 |
|  | 138 | account.mp | 509680 |
|  | 139 | biographies.mp | 1822 |
|  | 140 | stories.mp | 16832 |

1. **Medline on 15/11/2022**

| **Search Domain** | **Term #** | **Search Term** | **Hit count (1929 total)** |
| --- | --- | --- | --- |
|  | 1 | adolescen*.mp | 2187269 |
|  | 2 | Young Adult/ | 930355 |
|  | 3 | Adolescent/ | 2105438 |
|  | 4 | boy*.mp | 161650 |
|  | 5 | Child/ | 1758162 |
|  | 6 | child*.mp | 2546313 |
|  | 7 | CYP.mp | 15990 |
|  | 8 | girl*.mp | 156532 |
|  | 9 | juvenil*.mp | 97681 |
|  | 10 | minor*.mp | 330916 |
|  | 11 | Minors/ | 2651 |
|  | 12 | p?ediatric*.mp | 440137 |
|  | 13 | pubescen*.mp | 2556 |
|  | 14 | prepubescen*.mp | 1064 |
|  | 15 | pupil*.mp | 34926 |
|  | 16 | school*.mp | 340480 |
|  | 17 | student*.mp | 341448 |
|  | 18 | highschool.mp | 42 |
|  | 19 | Students/ | 64785 |
|  | 20 | teen*.mp | 32399 |
|  | 21 | young.mp | 1359876 |
|  | 22 | youth*.mp | 88876 |
|  | 23 | **AND** |  |
| Phenomenon of interest | 24 | COVID-19/ | 90407 |
|  | 25 | COVID*.mp | 150068 |
|  | 26 | coronavirus.mp | 96732 |
|  | 27 | SARS-CoV-2/ | 70168 |
|  | 28 | SARS-CoV2.mp | 2145 |
|  | 29 | SARSCoV2.mp | 44 |
|  | 30 | SARSCoV-2.mp | 183 |
|  | 31 | Sars adj coronavirus adj 2.mp |  |
|  | 32 | nCoV adj2 2019.mp | 1771 |
|  | 33 | 2019nCoV.mp | 11 |
|  | 34 | 2019-novel adj CoV.mp | 8 |
|  | 35 | nCoV adj 2019.mp |  |
|  | 36 | nCoV adj2 19.mp | 215 |
|  | 37 | severe adj acute adj respiratory adj syndrome adj coronavirus adj 2.mp |  |
|  | 38 | novel adj coronavirus.mp |  |
|  | 39 | novel adj corona adj virus.mp | 309 |
|  | 40 | corona adj virus adj disease adj 2019.mp | 634 |
|  | 41 | coronavirus adj disease adj 2019.mp |  |
|  | 42 | "novel coronavirus pneumonia".mp |  |
|  | 43 | "novel corona virus pneumonia".mp |  |
|  | 44 | quarantin*.mp | 11697 |
|  | 45 | self-isolat*.mp | 720 |
|  | 46 | lockdown.mp | 7376 |
|  | 47 | pandemic.mp | 93183 |
|  | 48 | lock adj down*.mp | 322 |
|  | 49 | dismiss* adj2 class*.mp | 31 |
|  | 50 | dismiss* adj2 school*.mp | 34 |
|  | 51 | school adj2 closure*.mp | 718 |
|  | 52 | social adj distanc*.mp | 6378 |
|  | 53 | shut-down*.mp | 1741 |
|  | 54 | shutdown*.mp | 2373 |
|  | 55 | stay* adj2 home.mp | 2655 |
|  | 56 | **AND** |  |
| Phenomenon of interest | 57 | (mental adj (health or ill-health or illness)).mp | 232992 |
|  | 58 | Mental Health/ | 232992 |
|  | 59 | agoraphobi*.mp | 4278 |
|  | 60 | wellbeing.mp | 20065 |
|  | 61 | well-being.mp | 87717 |
|  | 62 | psycho*.mp | 1937011 |
|  | 63 | Quality of Life/ | 215981 |
|  | 64 | "quality of life".mp | 378703 |
|  | 65 | QoL.mp | 43122 |
|  | 66 | emotion*.mp | 243003 |
|  | 67 | resilien*.mp | 41942 |
|  | 68 | challeng*.mp | 926866 |
|  | 69 | stress*.mp | 1082607 |
|  | 70 | cope.mp | 38450 |
|  | 71 | coping.mp | 59972 |
|  | 72 | Anxiety/ | 89064 |
|  | 73 | anx*.mp | 275453 |
|  | 74 | Mental Disorders/ | 167998 |
|  | 75 | depress*.mp | 577031 |
|  | 76 | low adj mood.mp | 898 |
|  | 77 | affective adj symptom*.mp | 15350 |
|  | 78 | dysthymi*.mp | 3667 |
|  | 79 | psychiatric.mp | 297889 |
|  | 80 | neurotic adj disorder*.mp | 18403 |
|  | 81 | panic.mp | 16996 |
|  | 82 | phobi*.mp | 18194 |
|  | 83 | dysphoric adj disorder*.mp | 1099 |
|  | 84 | affective adj disorder*.mp | 19713 |
|  | 85 | cyclothymic adj disorder*.mp | 805 |
|  | 86 | trauma*.mp | 447893 |
|  | 87 | trauma and stressor related disorders/ | 101 |
|  | 88 | adjustment adj disorder*.mp | 5530 |
|  | 89 | mood*.mp | 91052 |
|  | 90 | PTSD.mp | 27763 |
|  | 91 | posttrauma*.mp | 40664 |
|  | 92 | post-trauma*.mp | 63084 |
|  | 93 | distress*.mp | 156189 |
|  | 94 | grief.mp | 12979 |
|  | 95 | grieving.mp | 1658 |
|  | 96 | self-injur*.mp | 11029 |
|  | 97 | self-harm*.mp | 6914 |
|  | 98 | attention adj deficit.mp | 43169 |
|  | 99 | attention deficit hyperactivity disorder/ | 30702 |
|  | 100 | ADHD.mp | 27520 |
|  | 101 | hyperactiv*.mp | 73076 |
|  | 102 | disruptive adj behavio?r*.mp | 589 |
|  | 103 | SAD.mp | 10589 |
|  | 104 | fear*.mp | 100308 |
|  | 105 | frustrat*.mp | 20417 |
|  | 106 | happiness.mp | 10083 |
|  | 107 | happy.mp | 10253 |
|  | 108 | lonel*.mp | 9991 |
|  | 109 | self-esteem.mp | 22181 |
|  | 110 | self-mutilat*.mp | 4030 |
|  | 111 | Self-Injurious Behavior/ | 8470 |
|  | 112 | body adj image.mp | 23425 |
|  | 113 | suicid*.mp | 101347 |
|  | 114 | suicide/ | 41071 |
|  | 115 | self-killing*.mp | 63 |
|  | 116 | **AND** |  |
| Design | 117 | qualitative.mp | 276631 |
|  | 118 | participatory.mp | 17172 |
|  | 119 | interview*.mp | 420946 |
|  | 120 | focus adj group*.mp | 57757 |
|  | 121 | case adj stud*.mp | 115884 |
|  | 122 | mixed-method*.mp | 27640 |
|  | 123 | grounded adj theory.mp | 12913 |
|  | 124 | interpretative adj phenomenological adj analysis.mp | 1874 |
|  | 125 | semi-structured.mp | 51098 |
|  | 126 | IPA.mp | 6064 |
|  | 127 | unstructured.mp | 10677 |
|  | 128 | informal.mp | 25877 |
|  | 129 | in-depth.mp | 79172 |
|  | 130 | indepth.mp | 825 |
|  | 131 | face-to-face.mp | 32095 |
|  | 132 | structured.mp | 182083 |
|  | 133 | guide.mp | 195681 |
|  | 134 | ethnograph*.mp | 11950 |
|  | 135 | fieldwork.mp | 3790 |
|  | 136 | field adj work.mp | 1450 |
|  | 137 | key adj informant*.mp | 8580 |
|  | 138 | narrative.mp | 47546 |
|  | 139 | account.mp | 377669 |
|  | 140 | biographies.mp | 10652 |
|  | 141 | stories.mp | 13447 |

1. **PsycInfo on 15/11/2022**

| **Search Domain** | **Term #** | **Search Term** | **Hit count (452 total)** |
| --- | --- | --- | --- |
| Sample | 1 | adolescen*.mp | 486059 |
|  | 2 | Young Adult/ | 4090 |
|  | 3 | boy*.mp | 80074 |
|  | 4 | child*.mp | 834988 |
|  | 5 | CYP.mp | 554 |
|  | 6 | girl*.mp | 76717 |
|  | 7 | juvenil*.mp | 39986 |
|  | 8 | minor*.mp | 88462 |
|  | 9 | p?ediatric*.mp | 53129 |
|  | 10 | pubescen*.mp | 511 |
|  | 11 | prepubescen*.mp | 416 |
|  | 12 | pupil*.mp | 28082 |
|  | 13 | school*.mp | 483180 |
|  | 14 | student*.mp | 678896 |
|  | 15 | highschool.mp | 81 |
|  | 16 | Students/ | 678896 |
|  | 17 | teen*.mp | 24511 |
|  | 18 | young.mp | 222961 |
|  | 19 | youth*.mp | 115495 |
|  | 20 | **AND** |  |
| Phenomenon of interest | 21 | COVID-19/ | 1610 |
|  | 22 | COVID*.mp | 6868 |
|  | 23 | coronavirus.mp | 3894 |
|  | 24 | SARS-CoV-2/ | 2686 |
|  | 25 | SARS-CoV2.mp | 43 |
|  | 26 | SARSCoV2.mp | 3 |
|  | 27 | SARSCoV-2.mp | 9 |
|  | 28 | nCoV adj2 "2019".mp | 33 |
|  | 29 | 2019nCoV.mp | 0 |
|  | 30 | 2019-novel adj CoV.mp | 0 |
|  | 31 | nCoV adj2 19.mp | 0 |
|  | 32 | novel adj corona adj virus.mp | 10 |
|  | 33 | corona adj virus adj disease adj 2019.mp | 18 |
|  | 34 | quarantin*.mp | 786 |
|  | 35 | self-isolat*.mp | 203 |
|  | 36 | lockdown.mp | 203 |
|  | 37 | pandemic.mp | 7132 |
|  | 38 | lock adj down*.mp | 42 |
|  | 39 | dismiss* adj2 class*.mp | 81 |
|  | 40 | dismiss* adj2 school*.mp | 22 |
|  | 41 | school adj2 closure*.mp | 179 |
|  | 42 | social adj distanc*.mp | 3859 |
|  | 43 | shut-down*.mp | 291 |
|  | 44 | shutdown*.mp | 247 |
|  | 45 | stay* adj2 home.mp | 1197 |
|  | 46 | **AND** |  |
| Phenomenon of interest | 47 | (mental adj (health or ill-health or illness)).mp | 258370 |
|  | 48 | Mental Health/ | 72184 |
|  | 49 | agoraphobi*.mp | 6176 |
|  | 50 | wellbeing.mp | 16181 |
|  | 51 | well-being.mp | 100746 |
|  | 52 | psycho*.mp | 1432055 |
|  | 53 | Quality of Life/ | 42904 |
|  | 54 | "quality of life".mp | 93407 |
|  | 55 | QoL.mp | 11256 |
|  | 56 | emotion*.mp | 441684 |
|  | 57 | resilien*.mp | 38510 |
|  | 58 | challeng*.mp | 263249 |
|  | 59 | stress*.mp | 324161 |
|  | 60 | cope.mp | 30600 |
|  | 61 | coping.mp | 93355 |
|  | 62 | Anxiety/ | 30600 |
|  | 63 | anx*.mp | 266483 |
|  | 64 | Mental Disorders/ | 88367 |
|  | 65 | depress*.mp | 388508 |
|  | 66 | low adj mood.mp | 764 |
|  | 67 | affective adj symptom*.mp | 2266 |
|  | 68 | dysthymi*.mp | 4350 |
|  | 69 | psychiatric.mp | 240517 |
|  | 70 | neurotic adj disorder*.mp | 979 |
|  | 71 | panic.mp | 17978 |
|  | 72 | phobi*.mp | 22692 |
|  | 73 | dysphoric adj disorder*.mp | 931 |
|  | 74 | affective adj disorder*.mp | 34977 |
|  | 75 | cyclothymic adj disorder*.mp | 319 |
|  | 76 | trauma*.mp | 128866 |
|  | 77 | adjustment adj disorder*.mp | 2309 |
|  | 78 | mood*.mp | 90640 |
|  | 79 | PTSD.mp | 39172 |
|  | 80 | posttrauma*.mp | 48975 |
|  | 81 | post-trauma*.mp | 18711 |
|  | 82 | distress*.mp | 83617 |
|  | 83 | grief.mp | 19941 |
|  | 84 | grieving.mp | 3111 |
|  | 85 | self-injur*.mp | 10657 |
|  | 86 | self-harm*.mp | 7034 |
|  | 87 | attention adj deficit.mp | 37456 |
|  | 88 | ADHD.mp | 31135 |
|  | 89 | hyperactiv*.mp | 48441 |
|  | 90 | disruptive adj behavio?r*.mp | 678 |
|  | 91 | SAD.mp | 9355 |
|  | 92 | fear*.mp | 93871 |
|  | 93 | frustrat*.mp | 19806 |
|  | 94 | happiness.mp | 20137 |
|  | 95 | happy.mp | 12673 |
|  | 96 | lonel*.mp | 13636 |
|  | 97 | self-esteem.mp | 54981 |
|  | 98 | self-mutilat*.mp | 2424 |
|  | 99 | Self-Injurious Behavior/ | 2424 |
|  | 100 | body adj image.mp | 2424 |
|  | 101 | suicid*.mp | 73507 |
|  | 102 | suicide/ | 28404 |
|  | 103 | self-killing*.mp | 36 |
|  | 104 | **AND** |  |
| Design | 105 | qualitative.mp | 185399 |
|  | 106 | participatory.mp | 13325 |
|  | 107 | interview*.mp | 435749 |
|  | 108 | focus adj group*.mp | 39662 |
|  | 109 | case adj stud*.mp | 106798 |
|  | 110 | mixed-method*.mp | 28120 |
|  | 111 | grounded adj theory.mp | 17340 |
|  | 112 | interpretative adj phenomenological adj analysis.mp | 3324 |
|  | 113 | semi-structured.mp | 48471 |
|  | 114 | IPA.mp | 2429 |
|  | 115 | unstructured.mp | 5722 |
|  | 116 | informal.mp | 28109 |
|  | 117 | in-depth.mp | 55085 |
|  | 118 | indepth.mp | 542 |
|  | 119 | face-to-face.mp | 24623 |
|  | 120 | structured.mp | 147734 |
|  | 121 | guide.mp | 80780 |
|  | 122 | ethnograph*.mp | 31246 |
|  | 123 | fieldwork.mp | 7068 |
|  | 124 | field adj work.mp | 1049 |
|  | 125 | key adj informant*.mp | 3742 |
|  | 126 | narrative.mp | 52218 |
|  | 127 | account.mp | 144997 |
|  | 128 | biographies.mp | 2528 |
|  | 129 | stories.mp | 36994 |

1. **Web of Science on 15/11/2022**

| **Search Domain** | **Term #** | **Search Term (TS=)** | total hits 2129 |
| --- | --- | --- | --- |
| Sample | 1 | adolescen* | 54108 |
|  | 2 | boy* | 14420 |
|  | 3 | child* | 176401 |
|  | 4 | CYP | 1371 |
|  | 5 | girl* | 15862 |
|  | 6 | juvenil* | 13020 |
|  | 7 | minor* | 44706 |
|  | 8 | p$ediatric* | 52950 |
|  | 9 | *pubescen* | 758 |
|  | 10 | pupil* | 4341 |
|  | 11 | *school* | 69561 |
|  | 12 | student* | 97465 |
|  | 13 | teen* | 3771 |
|  | 14 | young | 102796 |
|  | 15 | youth* | 23019 |
|  | 16 | **AND** |  |
| Phenomenon of interest | 17 | COVID* | 138842 |
|  | 18 | coronavirus | 58674 |
|  | 19 | SARS-CoV* | 43045 |
|  | 20 | SARSCoV* | 595 |
|  | 21 | "2019 nCoV" | 1638 |
|  | 22 | 2019nCoV | 17 |
|  | 23 | "2019-novel CoV" | 6 |
|  | 24 | "nCoV 2019" | 50 |
|  | 25 | "nCoV 19" | 100 |
|  | 26 | "novel corona virus disease" | 57 |
|  | 27 | "corona virus disease 2019" | 605 |
|  | 28 | "novel corona virus pneumonia" | 8 |
|  | 29 | quarantin*.mp | 5194 |
|  | 30 | self-isolat* | 8597 |
|  | 31 | lockdown | 8597 |
|  | 32 | pandemic | 77736 |
|  | 33 | "lock down*" | 377 |
|  | 34 | "class dismiss**" | 1 |
|  | 35 | "dismiss* class*" | 2 |
|  | 36 | "school dismissal*" | 3 |
|  | 37 | "dismiss* school*" | 1 |
|  | 38 | "close* school*" | 54 |
|  | 39 | "school closure*" | 600 |
|  | 40 | "social distanc*" | 5659 |
|  | 41 | "closure school*" | 1 |
|  | 42 | shut-down* | 681 |
|  | 43 | shutdown* | 1650 |
|  | 44 | "stay* at home" | 1204 |
|  | 45 | **AND** |  |
| Phenomenon of interest | 46 | "mental health" | 43291 |
|  | 47 | agoraphobi* | 178 |
|  | 48 | wellbeing | 8815 |
|  | 49 | well-being | 24826 |
|  | 50 | psycho* | 113071 |
|  | 51 | "mental* ill*" | 6573 |
|  | 52 | "quality of life" | 60009 |
|  | 53 | QoL | 6766 |
|  | 54 | emotion* | 54454 |
|  | 55 | resilien* | 30196 |
|  | 56 | challeng* | 311006 |
|  | 57 | stress* | 242299 |
|  | 58 | cope | 23170 |
|  | 59 | coping | 23232 |
|  | 60 | anx* | 47540 |
|  | 61 | "mental disorder*" | 7023 |
|  | 62 | depress* | 71869 |
|  | 63 | "low mood" | 177 |
|  | 64 | "affective symptom*" | 282 |
|  | 65 | dysthymi* | 110 |
|  | 66 | psychiatric | 18657 |
|  | 67 | "neurotic disorder*" | 27 |
|  | 68 | panic | 1977 |
|  | 69 | phobi* | 1312 |
|  | 70 | "dysphoric disorder*" | 138 |
|  | 71 | "affective disorder*" | 1089 |
|  | 72 | "cyclothymic disorder*" | 8 |
|  | 73 | *trauma* | 49439 |
|  | 74 | "adjustment disorder*" | 179 |
|  | 75 | mood* | 12433 |
|  | 76 | PTSD | 5413 |
|  | 77 | distress* | 26567 |
|  | 78 | grief | 1918 |
|  | 79 | grieving | 346 |
|  | 80 | self-inj* | 1291 |
|  | 81 | self-harm* | 1665 |
|  | 82 | "attention deficit" | 4576 |
|  | 83 | ADHD | 4461 |
|  | 84 | hyperactiv* | 8054 |
|  | 85 | "disruptive behavio?r*" | 89 |
|  | 86 | SAD | 1727 |
|  | 87 | fear* | 19393 |
|  | 88 | frustrat* | 4392 |
|  | 89 | happiness | 3786 |
|  | 90 | happy | 2463 |
|  | 91 | lonel* | 3905 |
|  | 92 | self-esteem | 5012 |
|  | 93 | self-mutilat* | 71 |
|  | 94 | "body image" | 2355 |
|  | 95 | suicid* | 11900 |
|  | 96 | self-killing* | 8 |
|  | 97 | **AND** |  |
| Design | 98 | qualitative | 82936 |
|  | 99 | participatory | 8134 |
|  | 100 | interview* | 86017 |
|  | 101 | "focus group*" | 13250 |
|  | 102 | "case stud*" | 77970 |
|  | 103 | mixed-method* | 13350 |
|  | 104 | "grounded theory" | 3393 |
|  | 105 | "interpretative phenomenological analysis" | 745 |
|  | 106 | IPA | 1628 |
|  | 107 | informal | 9967 |
|  | 108 | in-depth | 32056 |
|  | 109 | indepth | 344 |
|  | 110 | face-to-face | 9341 |
|  | 111 | *structured | 70443 |
|  | 112 | guide | 96044 |
|  | 113 | ethnograph* | 9198 |
|  | 114 | fieldwork | 3809 |
|  | 115 | "field work" | 738 |
|  | 116 | "key informant*" | 2239 |
|  | 117 | narrative | 31485 |
|  | 118 | account. | 148786 |
|  | 119 | biographies | 2174 |
|  | 120 | stories | 19286 |

1. **MedRXIV**

| **Search 1 07/07/21:** |  |
| --- | --- |
| TI: covid coronavirus pandemic lockdown quarantine  AB TI: adolescent adolescents young youth child boy girl  FULL: mental depress anxiety anxious | Limit to 01/01/2020 to 07/07/2021 and sort results by best match  1000 results screened until 10x consecutive irrelevant results  Imported relevant results to Covidence |
| **Search 2 07/07/21:** |  |
| covid child mental qualitative | Limit to 01/01/2020 to 07/07/2021 and sort results by best match  50 results screened until 10x consecutive irrelevant results  Imported relevant results to Covidence |
| **Search 3 07/07/21:** |  |
| coronavirus young mental qualitative | Limit to 01/01/2020 to 07/07/2021 and sort results by best match  60 results screened until 10x consecutive irrelevant results  Imported relevant results to Covidence |
| **Search 4 07/07/21:** |  |
| covid adolescents mental qualitative | Limit to 01/01/2020 to 07/07/2021 and sort results by best match  25 results screened until 10x consecutive irrelevant results  Imported relevant results to Covidence |
| **Search 5 15/11/22:** |  |
| TI: covid coronavirus pandemic lockdown quarantine  AB TI: adolescent adolescents young youth child boy girl  FULL: mental depress anxiety anxious | Limit to 07/07/2021 to 15/11/2022 and sort results by best match  266 results screened until 10x consecutive irrelevant results  Imported relevant results to Covidence |

1. **Google**

| **Search 1 11/08/21:** |  |
| --- | --- |
| (young or youth or child or adolescent or teen or student or school) and (COVID or coronavirus or lockdown or pandemic) and (anxiety or depression or psych or mental) and (qualitative or focus group or account or interview) | 87 results in total (excluding hits that Google filters out as they're irrelevant)  All screened |
| **Search 2 11/08/21:** |  |
| covid lockdown mental health young people adolescents qualitative | 31500000 results screened until 20x consecutive irrelevant results (63 screened) |
| **Search 3 12/12/22:** |  |
| (young or youth or child or adolescent or teen or student or school) and (COVID or coronavirus or lockdown or pandemic) and (anxiety or depression or psych or mental) and (qualitative or focus group or account or interview) | 23 results in total (excluding hits that Google filters out as they’re irrelevant)  All screened |
| **Search 4 12/12/22:** |  |
| covid lockdown mental health young people adolescents qualitative | 1320000 results screened until 20x consecutive irrelevant results (97 screened) |

1. **OSF**

| **Search 1 7/7/21:** |  |
| --- | --- |
| (young OR adolescen* OR boy* OR girl* OR child OR CYP OR juvenil* OR minor* OR p?ediatric* OR pubescen* OR prepubescen* OR pupil* OR student* OR teen* OR youth*) AND (COVID* OR SARS-CoV* OR SARSCoV* OR coronavirus OR nCoV OR CoV OR "corona virus" OR quarantin* OR self-isolat* OR lockdown* OR pandemic OR "lock down*" OR "class dismiss*" OR "dismiss* class*" OR "school dismiss*" OR "dismiss* school" OR "school closure*" OR "closed school*" OR "social distanc*" OR "stay* at home" OR shut-down* OR shutdown*) AND ("mental health" OR agoraphobi* OR wellbeing OR well-being OR psycho* OR "mental ill*" OR "quality of life" OR QoL OR emotion* OR resilien* OR challeng* OR stress* OR cope OR coping OR anx* OR "mental disorder*" OR depress* OR "low mood" OR "affective symptom" OR dysthymi* OR psychiatric OR "neurotic disorder*" OR panic OR phobia* OR "dysphoric disorder*" OR "affective disorder*" OR "cyclothymic disorder*" OR trauma* OR "adjustment disorder*" OR mood* OR PTSD OR posttrauma* OR post-trauma* OR distress* OR grief OR grieving OR self-inj* OR self-harm* OR "attention deficit" OR ADHD OR hyperactiv* OR "disruptive behaviour*" OR SAD OR fear* OR frustrat* OR happiness OR happy OR lonel* OR self-esteem OR self-mutilat* OR "body image" OR suicid* OR self-killing*) AND (qualitative OR participatory OR interview* OR "focus group*" OR "case stud*" OR mixed-method* OR "grounded theory" OR "interpretative phenomenological analysis" OR semi-structured OR IPA OR unstructured OR informal OR in-depth OR indepth OR face-to-face OR structured OR guide OR ethnograph* OR fieldwork OR "field work" OR "key informant*" OR narrative OR account OR biographies OR stories) | Filtered to psychology; mental and social health; psychiatry and psychology.  47 results in total  All screened |
| **Search 2 15/11/22:** |  |
| (young OR adolescen* OR boy* OR girl* OR child OR CYP OR juvenil* OR minor* OR p?ediatric* OR pubescen* OR prepubescen* OR pupil* OR student* OR teen* OR youth*) AND (COVID* OR SARS-CoV* OR SARSCoV* OR coronavirus OR nCoV OR CoV OR "corona virus" OR quarantin* OR self-isolat* OR lockdown* OR pandemic OR "lock down*" OR "class dismiss*" OR "dismiss* class*" OR "school dismiss*" OR "dismiss* school" OR "school closure*" OR "closed school*" OR "social distanc*" OR "stay* at home" OR shut-down* OR shutdown*) AND ("mental health" OR agoraphobi* OR wellbeing OR well-being OR psycho* OR "mental ill*" OR "quality of life" OR QoL OR emotion* OR resilien* OR challeng* OR stress* OR cope OR coping OR anx* OR "mental disorder*" OR depress* OR "low mood" OR "affective symptom" OR dysthymi* OR psychiatric OR "neurotic disorder*" OR panic OR phobia* OR "dysphoric disorder*" OR "affective disorder*" OR "cyclothymic disorder*" OR trauma* OR "adjustment disorder*" OR mood* OR PTSD OR posttrauma* OR post-trauma* OR distress* OR grief OR grieving OR self-inj* OR self-harm* OR "attention deficit" OR ADHD OR hyperactiv* OR "disruptive behaviour*" OR SAD OR fear* OR frustrat* OR happiness OR happy OR lonel* OR self-esteem OR self-mutilat* OR "body image" OR suicid* OR self-killing*) AND (qualitative OR participatory OR interview* OR "focus group*" OR "case stud*" OR mixed-method* OR "grounded theory" OR "interpretative phenomenological analysis" OR semi-structured OR IPA OR unstructured OR informal OR in-depth OR indepth OR face-to-face OR structured OR guide OR ethnograph* OR fieldwork OR "field work" OR "key informant*" OR narrative OR account OR biographies OR stories) | Filtered to psychology; mental and social health; psychiatry and psychology.  70 results in total  All screened |
