# Supplementary material for: The impact of COVID-19 on young people’s mental health, wellbeing and routine from a European perspective: A co-produced qualitative systematic review
Source: PLoS One. 2024 Mar 20;19(3):e0299547. doi: 10.1371/journal.pone.0299547 (PMC10954119; doi:10.1371/journal.pone.0299547)
Supplement: S1 Table — (DOCX) [file pone.0299547.s004.docx]

**S4 Fig.: Study characteristics.**

| **First author, year** | **Country** | **Wave 1, 2 or 3 (dates during data generation)** | **Qualitative methods/ analysis** | **Population** | **Sample size** | **Theoretical or conceptual basis underlying study** | **Primary source of data** |
| --- | --- | --- | --- | --- | --- | --- | --- |
| Abawi (2020)[39] | The Netherlands | Wave 1 (04/2020) | SSI/TA | Attended an obesity centre, (52% female; median age 10.5 years) | 75 | Grounded theory | Passages from documentation of telephone interviews |
| Ashworth (2022)[40] | UK | Wave 2 (09/2020-12/2020) | IDI/RTA | Any YP (36% female; mean age 12; SD N/A) | 14 | N/A | Quotes |
| Barnardos (2020a) | UK | Not reported | I/U | Marginalised groups (with existing mental health difficulties; BAME CYP; poverty; young people entering education, employment or training; CYP with special educational needs; young carers; young people leaving care; LGBTQ+ YP; children with a parent in custody) | 150 | Co-production/peer led | Statistics and quotations presented in the report. |
| Barnardos (2020b) | UK | Wave 1 (06/05/2020-01/06/2020) | IDI/TA | Any YP (55% female; age range 13-25) | 113 | Grounded in listening to voices of young people | Quotes |
| Bengtsson (2021) | Denmark | Not Reported | IDI/TA | Gaming experience (66% female; age range 16-20) | 35 | Social Practice theoretical framework | Quotes |
| Branquinho (2020)(Branquinho, Kelly, Arevalo, Santos, & Gaspar De Matos, 2020) | Portugal | Wave 1 (14/04/2020-18/05/2020) | ORS/U | Any YP (70% female; mean age 19.2 years) | 617 | Engel's Biopsychosocial model | Open-response survey questions; extracts included in manuscript as well as tabulated categories, sub-categories and key-ideas |
| Branquinho (2021)(Branquinho, Santos, Ramiro, & Gaspar De Matos, 2021) | Portugal | Wave 2 (15/10/2020-08/11/2020) | ORS/CA | Any YP (71% female; mean age 18.4 years SD 2.1) | 304 | Not reported | Quotes |
| Branquinho (2022) | Portugal | Wave 3 (02/2021-03/2021) | ORS/CA | Any YP (71% female; mean age 19 years) | 592 | Not reported | Quotes |
| Burgess (2022) | UK | Wave 1 | FGD/TA | YP minoritised by race (80% female; age range 16-25) | 40 | Co-production | Quotes |
| Cage (2021) | UK | Wave 2 (11/2021-12/2021) | ORS/CA | Autistic and non-autistic university students (83% female; age not reported; | 385 | Not reported | Quotes |
| Carers Trust (2020) | UK | Wave 1 (06/2020) Relaxing of Lockdown 1 | ORS/TA | Carers (gender not Reported; age range 12-25) | 961 | Not reported | Quotes |
| Cerovic (2021) | Serbia | Wave 1 (05/2020-06/2020)  When the state of emergency was lifted | MGN/TA | School children (67% female; age range 7-18) | 45 | Not reported | Quotes from MGN (36 stories, 31 letters, and 39 suggestions for teachers about distance learning) |
| Children's Commissioner for Wales (2020) | UK | Wave 1 (05/2020) Lockdown 1 | ORS/CA | Any YP (56% female; age range 7-18) | 23,488 | Not reported | Quotes |
| Children’s Commission for Wales (2021) | UK | Wave 1 (01/02/2021) | ORS/MGN | Any YP (age range 3-18) | 19,737 | Not reported | Quotes |
| Children's Commissioner for England (2020)(England, 2020) | UK | Wave 2 (05/10/2020-11/10/2020) Pupils had just returned to school | ORS/U | School children (gender: 50% female; age range 8-17) | 1,500 | Not reported | Quotes |
| Collaco (2021) | UK | Wave 1 (05/2020-07/2020) | ORS/CA | YP with cystic fibrosis (gender: 70.7% female) | 99 | Not reported | Quotes |
| Dedryver (2021) | UK | Not Reported | SSI/Ta | Any YP aged 18-24 (gender: 73% female; mean age 22, SD Not Reported) | 15 | Not reported | Quotes |
| Demkowicz (2022) | UK | Wave 1 (N/A) Lockdown 1 | NAC/TA | Any YP (gender: 80% female; age range 16-19) | 109 | Not reported |  |
| Dewa (2021) | UK | Wave 1 (23/04/2020-28/05/2020) Lockdown 1 | SSI/CTA | Any YP (gender: 78% female; mean age 20.8) | 18 | Co-production | Quotes |
| Dunlop (2021) | UK | Not Reported | SSI/TA | YP who identify as bisexual and have experience of non-suicidal self-injury (NSSI) (gender: 53% cisgender, 27% non-binary/third gender, 13% cisgender man, 1% transgender man; age range 16-25) | 15 | Critical realist epistemological perspective | Quotes |
| Essex Community Foundation (2020) | UK | N/A (04/2020-06/2020) | C/U | YP from voluntary youth settings (gender: not reported; age range 9-25) | 202 | Not reported | Quotes from individuals within youth sessions |
| Fioretti (2020) | Italy | Wave 1 (04/2020) Lockdown 1 | NAC/TA | Any YP (gender: females 74.8%; mean age 16.6 SD 1.4) | 2758 | Autonomy acquisition during adolescence; biographical disruption | Quotes from individuals |
| Giannakopoulos (2021) | Greece | Wave 1 (04/2020) | IDI/TA | Psychiatric inpatients (gender: not reported, age range 12-17) | 9 | Not reported | Quotes |
| Girlguiding (2020) | UK | Wave 1 (05/2020) Lockdown 1 | ORS/TA | Young women, age range 4-18 | 6678 | Not reported | Quotes from individuals |
| Giusti (2020) | Italy | Wave 1 (03/2020-05/2020)  Lockdown 1 | ND/NA | University students that are users of a University-provided digital counselling service (gender: 81.6% female; mean age 22.5 SD 3.3) | 103 | Not reported | Passages from narrative diary |
| Government of Ireland Department of Children and Youth Affairs and SuponOut.ie (2020) | Ireland | Wave 1 and 2 (Late June/early July 2020) | ORS/U | Any YP (gender: 51% female; age range 15-24) | 2173 | Not reported | Quotes |
| Griffin (2022) | UK | Wave 3 (02/2021-04/2021) | SSI/TA | Nursing students (gender: 86.7%; age range 19-23) | 15 | Not reported | Quotes |
| Hamilton (2020) | UK | Wave 1 (01/05/2020) Lockdown 1 | FGD/U | School children (gender: 56% female; age range 10-16) | 25 | Peer led | Virtual whiteboards; quotes from individuals through group discussion |
| Hanghoj (2021) | Denmark | Wave 1 (13/03/2020-15/04/2020) Lockdown 1 | SSI/RTA | YP cancer patients and survivors aged 18-29 (gender N/A; mean age 23.5 SD N/A) | 13 | Not reported | Quotes |
| Healthwatch Richmond (2020) | UK | Wave 1 (18/05/2020-25/06/2020)  Mostly Lockdown 1 | ORS/U | Any YP (gender: approximately 2 in 3 respondents were female, 1 in 3 were male and six people  reported that they were transgender (2) or non-binary (4).; age range 13-25) | 346 | Not reported | Quotes |
| Hosszu (2022) | Romania | Wave 2 (11/2020) | ORS/CA | Students (70% female; age not reported) | 5372 | Not reported | Keywords and quotes |
| Hughes (2022) | UK | Not reported | SSI/TA | Any YP (56% female; age range 12-16) | 9 | Not reported | Quotes |
| Huscsava (2021) | Austria | Wave 1  Initial restrictions started | SSI/TA | YP psychiatric patients aged 12-18 (gender 87% female; mean age 16.2 SD 1.6) | 30 | Not reported | Quotes |
| Jemini-Gashi (2022) | Kosovo | Wave 3 (04/2021-05/2021) | SSI/TA | Any YP (53% female; average age 17) | 30 | Not reported | Quotes |
| Jenholt-Nolbris (2022) | Sweden | Wave 2 (07/2020-11/2020) | SSI/TA | School children (age range 6-14) | 151 | Not reported | Quotes |
| Kelly (2021) | UK | Wave 2 (08/2020-09/2020) | SSI/TA | Care leavers aged 18-24; female: 17; male: 7; average age: 20 years-old. | 24 | Not reported | Quotes |
| Larcher (2020) | UK | Wave 1 (05/2020) Lockdown 1 | FGD/TA | Members of the Hospital Young People's Forum (gender not reported; age range 11-18 years) | 15 | Not reported | Quotes |
| Loer (2022) | Germany | Wave 3 (05/2021-06/2021) | FGD/CA | Any YP (age range 13-25) | 24 | Cognitive, behavioural, conative, and affective components of health literacy conceptual framework | Quotes |
| Lukoševičiūtė (2022) | Lithuania | Wave 3 (18/04/2021-22/07/2021) | SSI/TA | Any YP (age 11-17) | 19 | Not reported | Quotes |
| Maftei (2022) | Romania | Not reported | ORS/CA | Children and adolescents (gender: 56.1% female; aged 10-13) | 155 | Ekman’s theoretical framework | Quotes and individual words |
| Malmquist (2022) | Sweden | Not reported | OSI/TA | Young people identified as LGBTQ+ (age range 20–29 years) | 15 | Not reported | Quotes |
| McCluskey (2021) | UK | Wave 2 (08/2020-09/2020)  Pupils were just returning to school | FGD/TA | School children (gender; 71% female; age range 14-18) | 45 | Not reported | Quotes |
| McKinlay (2022) | UK | In-between Wave 1 and 2; Lockdown 1 and 2 (06/2020-01/2021) | SSI/TA | Any YP (gender: female: 23; male: 14; age range 13-24) | 37 | Not reported | Quotes from individuals |
| MCR Pathways (2020) | UK | In-between Wave 1 and 2 (01/06/2020-07/07/2020) During easing of lockdown restrictions | ORS/U | YP identified as disabled (gender not reported; age range 13-18) | 1,347 | Grounded in listening to voices of young people | Quotes from individuals |
| Mietola (2021) | Finland | Wave 1 (04/2020) Lockdown 1 | MGN/NA | YP identified as disabled (gender not reported; age not reported) | 14 | Not reported | Essays, photo compilations, poems, etc |
| Nolan (2020) | UK | Wave 1 (01/05/2020) Lockdown 1 | ORS/U | YP with experience of the criminal justice system (33% female; age range 20-25) | 70 | Not reported | Quotes from individuals |
| Northern Ireland Youth Forum (2020) | UK | Wave 2 (10/2020)  Circuit breaker lockdown restrictions | ORS/TA | Any YP (gender not reported; age range 11-25) | 1,065 | Not reported | Quotes |
| O’Kane (2021) | UK | Wave 1 (05/2020-06/2020) | ORS/TA | Participants of the Walking in Schools (WISH) study (100% female; mean age 13.8 years, SA not reported) | 16 | Not reported | Quotes |
| Olah (2022) | Hungary | Wave 6 (11/2021-12/2021) | SSI/CA | Medical students (gender: 73% males; mean age 21.8, ±1.88) | 26 | Theoretical framework of patients’ health care decision making | Quotes |
| Partnership for Young London (2020) | UK | Wave 1 (03/2020-05/2020) Lockdown 1 | ORS/TA | Any YP (51% female; age range 14-25) | 166 | Not reported | Quotes |
| Perming (2022) | Sweden | Wave 3 (05/2021-10/2021) | FGD/CA | Any YP (age range 16-19) years | 41 | Not reported | Quotes |
| Phillips (2022) | UK | 2020-21 | SSIP | Higher education students | 46 | Co-production | Quotes |
| Procentese (2021) | Italy | Wave 1 (25-31/03/2020, and 22-28/04/2020) | ND/GT | Any YP (89%; mean age 23.0 years, SD 0.9) | 36 | DeWolfe’s linear model of disaster responses/Grounded theory | Quotes |
| Riiser (2022) | Norway | Wave 2 (11/2020-12/2020)  Some restrictions beginning to increase by December | FGD/CA | Any YP (39% female; age range 9-15) | 17 | Health literacy and health-related quality of life used to provide directions for the analysis'; Built on an earlier piece of quantitative work from the team that identified lower quality of life and poor mental health later in the pandemic, seeks to offer greater depth | Quotes |
| Roberts (2020) | UK | Wave 1 (05/2020-07/2020) | SSI, FGD, MGN/TA | YP who left/leaving state care (81% female; age range 17-24) | 21 | Not reported | Participant quotes, visual materials and poetry submissions |
| Sachs (2020) | UK | Wave 2 (07/2020-08/2020) | SSI, FGD/U | YP who identify as LGBTQ+, Gypsy, Traveller, Roma or a carer (gender not reported; age range 10-35) | 31 | Not reported | Quotes |
| Sawyer (2022) | UK | Pre-Covid-19 (05/2019-08/2019) Wave 1 (06/2020-087/2020) | SSI/TA | Young adults in most deprived boroughs of London (age range 18-24 years) | 48 (pre-Covid-19) and 35 (Wave 1) | Social representations theory | Quotes/free association grid drawings |
| Scott (2021) | UK | Wave 2 (07/2020-10/2020)  During local tiers/restrictions | ND, SSI/TA | YP aged 13-17; female: 58% | 31 | Inductive approach to theory building | Quotes, Images |
| Scottish Youth Parliament (2020) | UK | N/A (10/2020-11/2020)  During phase 3 of relaxation of lockdown measures in Scotland | FGD/TA | YP minoritised by disability, ethnicity, care experienced, experience of the criminal justice system, and being a carer (gender not reported; age range 14-24) | 37 | Not reported | Quotes |
| Sica (2022) | Italy | Wave 3 (09/2021) | ORS/NCA | Students in their first year of social sciences degree from middle to high socioeconomic background (74% female; age range 18-21 years, mean 19.65 SD 2.28) | 70 | Narrative approach | Quotes |
| Stewart (2022) | UK | Wave 2 (08/2020 - 09/2020) | ORS/TA | Students in their first year of social sciences degree from middle to high socioeconomic background (74% female; age range 18-21 years, mean 19.65 SD 2.28) | 518 | Not reported | Quotes |
| StreetDoctors (2020) | UK | In-between Wave 1 and 2 (05/2020-06/2020) Lockdown 1 | ORS/TA | YP at risk of youth violence (39% female; age range 14-25) | 44 | Not reported | Quotes |
| The Children's Society (2020) | UK | Wave 1 Lockdown 1 to easing of Lockdown 1 | C/TA | Any YP (gender not reported; age range 8-19) | 150 | Not reported | Quotes |
| Thompson (2021) | UK | In-between Wave 1 and 2 (05/2020-July 2020) | SSI/PD/TA | Any children in England and Wales (61% female; aged 7-11 years) | 18 | Not reported | Drawings and quotes |
| Tishelman (2022) | Sweden | Wave 1 (04/2020-06/2020) | PD/SPA | School children in Sweden (41% male; aged 13-15 years old) | 187 | A qualitative, reconstructive research paradigm | Drawings |
| Torronen (2022) | Sweden | Wave 3 (09/2021-10/2021) | SSI/SMA | Young people living in Sweden (58% female; age 18-24 years, median 22) | 33 | Not reported | Quotes |
| Town (2021) | UK | Not reported | SSI, FGD/RTA | YP who identify as LGBTQ+ (60% female; mean age 1.3 SD 3.4) | 20 | Realist ontological and relativist epistemological stance; recognising that a reality independent from subjective experience exists, and situating findings in the belief that it is not possible to objectively understand or fully access this reality. This means researchers focused primarily on semantic or language-based themes, with some exploration of latent meanings | Quotes from individuals within focus groups |
| Tse (2021) | UK | Wave 1 05/2020-06/2020 Lockdown 1 | ORS/TA | YP with kidney conditions (gender not reported; median sample age 21.0 age range 12-30) | 118 | Not reported | Quotes |
| Tyrrell (2020) | UK | 08/2020-10/2020 Pupils were just returning to school | IDI/TA | Any YP (56% female; age range 12-19) | 16 | Participatory and child centered approach to empower young people | Quotes |
| Widnall (2022) | UK | Wave 3 and 4 (12/2020-03/2021) | FGD/SSI/TA | Adolescents in Year 10 at school in deprived part of England (66% female; aged 14-15 years) | 25 | Not reported | Quotes |
| Winter (2022) | UK | Wave 1 and 2 (05/2020-03/2021) | E/RTA | UK residents using social media (no gender or age information reported) | 1033 original posts and 13,860 associated comments | Interpretive qualitative framework of medical anthropology | Quotes |
| Wood (2021) | UK | Wave 3 (03/2021-05/2021) | FGD/TA | Children and young people across Scotland (56% female, aged 11-17 years) | 25 | Social Determination Theory | Quotes |
| Young Healthwatch Westminster (2020) | UK | Wave 1 (27/04/2020-16/06/2020)  Lockdown 1 to easing of Lockdown 1 | ORS/U | Any YP (gender not reported; age range 14-25) | 144 | Not reported | Quotes |
| YoungMinds (2020a) | UK | In-between Wave 1 and 2 (06/2020-07/2020)  Easing of Lockdown 1 | ORS/TA | YP who had accessed some form of mental health support in the first three months of the year (81% female; age range 13-25) | 2036 | Not reported | Quotes |
| YoungMinds (2020b) | UK | Wave 1 (09/2020)  Return to school after Lockdown 2 | ORS/TA | YP with a history of mental health needs who have returned to secondary school or sixth-form college (74% female; age range 11-18) | 2011 | Not reported | Quotes |
| YoungMinds (2020c) | UK | Wave 1 (20/03/2020 and 25/05/2020) Lockdown 1 Friday 20th March (the day that schools closed to most children) and Wednesday 25th March (when there had been a further tightening of restrictions) | ORS/TA | YP with a history of mental health needs who have returned to secondary school or sixth-form college (74% identified as female; age range 10-18 years) | 2,111 | Not reported | Quotes |
| YoungMinds (2021) | UK | Wave 3 (01/2021-02/2021) Lockdown 3 | ORS/TA | YP who had accessed some form of mental health support in the first three months of the year (79% female; age range 13-25) | 2438 | Not reported | Quotes |

**Key:** C Consultations, CA Content analysis, CTA Co-produced thematic analysis, E Ethnography, F Form, FGD Focus group discussions, GT Grounded Theory, IDI In depth interviews, LGBTQ+ Lesbian, Gay, Bisexual, Transgender, Queer and others, ORS Open-response survey questions, OSI Open-ended structured interviews, I Insights, SSI Semi-structured interviews, SSIP semi-structured interviews with peers, MGN Multi-genre narratives, NAC Narrative account, NCA Narrative coding approach, N/A Not applicable, ND Narrative diaries, PD Participatory drawings, RTA Reflexive thematic analysis, SA Socio-material analysis, SPA Serial picture analysis, SD Standard deviation, TA Thematic analysis, UK United Kingdom, YP Young people

Barnardo’s. (2020b). *Devalued by Forces Beyond Your Control: Experences of COVID-19 lockdown restrictions and visions for the future, from young people who are supported by Bardnardo’s*. Retrieved from Essex, UK:

Branquinho, C., Kelly, C., Arevalo, L. C., Santos, A., & Gaspar De Matos, M. (2020). “Hey, we also have something to say”: A qualitative study of Portuguese adolescents’ and young people's experiences under COVID‐19. *Journal of Community Psychology, 48*(8), 2740-2752. doi:10.1002/jcop.22453

Branquinho, C., Santos, A. C., Ramiro, L., & Gaspar De Matos, M. (2021). #COVID#BACKTOSCHOOL: Qualitative study based on the voice of Portuguese adolescents. *Journal of Community Psychology, 49*(7), 2209-2220. doi:10.1002/jcop.22670

England, C. s. C. o. (2020). *Some sort of normal*. Retrieved from London:

Hosszu, A., Rughiniş, C., Rughiniş, R., & Rosner, D. (2021). Webcams and Social Interaction During Online Classes: Identity Work, Presentation of Self, and Well-Being. *Frontiers in Psychology, 12*, 761427. doi:10.3389/fpsyg.2021.761427
