# Supplementary material for: The impact of COVID-19 on young people’s mental health, wellbeing and routine from a European perspective: A co-produced qualitative systematic review
Source: PLoS One. 2024 Mar 20;19(3):e0299547. doi: 10.1371/journal.pone.0299547 (PMC10954119; doi:10.1371/journal.pone.0299547)
Supplement: S2 Table — (DOCX) [file pone.0299547.s005.docx]

Supplementary file 5: Mixed Methods Appraisal Tool Results (journal articles only)

|  |  | **SCREENING** | | **1. QUALITATIVE STUDIES** | | | | | **5. MIXED METHODS STUDIES** | | | | | Overall quality score |
| --- | --- | --- | --- | --- | --- | --- | --- | --- | --- | --- | --- | --- | --- | --- |
|  |  | **QUESTIONS** | |  |  |  |  |  |  |  |  |  |  |  |
| First | Year | S1. Are there clear research questions [or aim, objective]? | S2. Do the collected data allow to address the research questions [aim, objective(s)]? | 1.1. Is the qualitative approach appropriate to answer the research question? | 1.2. Are the qualitative data collection methods adequate to address the research question? | 1.3. Are the findings adequately derived from the data? | 1.4. Is the interpretation of results sufficiently substantiated by data? | 1.5. Is there coherence between qualitative data sources, collection, analysis and interpretation? | 5.1. Is there an adequate rationale for using a mixed methods design to address the research question? | 5.2. Are the different components of the study effectively integrated to answer the research question? | 5.3. Are the outputs of the integration of qualitative and quantitative components adequately interpreted? | 5.4. Are divergences and inconsistencies between quantitative and qualitative results adequately addressed? | 5.5. Do the different components of the study adhere to the quality criteria of each tradition of the methods involved? |  |
| Author |  |  |  |  |  |  |  |  |  |  |  |  |  |  |
| Abawi | 2020 | 1 | 1 |  |  |  |  |  | 1 | 1 | 1 | 1 | 1 | **** |
| Ashworth | 2021 | 1 | 1 | 1 | 1 | 1 | 1 | 1 |  |  |  |  |  | **** |
| Bengtsson | 2021 | 1 | 1 | 1 | 1 | 1 | 1 | 1 |  |  |  |  |  | **** |
| Branquinho (a) | 2020 | 1 | 1 |  |  |  |  |  | 0 | 1 | 1 | 0 | 0 | * |
| Branquinho (b) | 2021 | 1 | 1 |  |  |  |  |  | 1 | 1 | 1 | 1 | 0 | *** |
| Branquinho (c) | 2022 | 1 | 1 |  |  |  |  |  | 1 | 1 | 1 | Can't tell | 1 | *** |
| Burgess | 2021 | 1 | 1 | 1 | 1 | 1 | 1 | 1 |  |  |  |  |  | **** |
| Cage | 2021 | 1 | 1 |  |  |  |  |  | 1 | 1 | 1 | 1 | 1 | **** |
| Cerovic | 2021 | 1 | 1 | 1 | 1 | N/A | N/A | N/A |  |  |  |  |  | * |
| Collaco | 2021 | 1 | 1 |  |  |  |  |  | 1 | 1 | 1 | 1 | 1 | **** |
| Dedryver | 2020 | 1 | 1 | 1 | 1 | 1 | 1 | 1 |  |  |  |  |  | **** |
| Dewa | 2021 | 1 | 1 |  |  |  |  |  | 1 | 1 | 1 | 1 | 1 | **** |
| Dunlop | 2021 | 1 | 1 | 1 | 1 | 1 | 1 | 1 |  |  |  |  |  | **** |
| Fioretti | 2020 | 1 | 1 | 1 | 1 | 1 | 1 | 1 |  |  |  |  |  | **** |
| Giannakopoulos | 2021 | 1 | 1 | 1 | 1 | 1 | 1 | 1 |  |  |  |  |  | **** |
| Giusti | 2020 | 1 | 1 |  |  |  |  |  | 1 | 1 | 1 | 1 | 0 | *** |
| Griffin | 2022 | 1 | 1 | 1 | 1 | 1 | 1 | 1 |  |  |  |  |  | **** |
| Hanghoj | 2021 | 1 | 1 | 1 | 1 | 1 | 1 | 1 |  |  |  |  |  | **** |
| Hosszu | 2022 | 1 | 1 | 1 | 1 | 1 | 1 | 1 |  |  |  |  |  | **** |
| Hughes | 2022 | 1 | 1 | 1 | 1 | 1 | 1 | 1 |  |  |  |  |  | **** |
| Huscsava | 2021 | 1 | 1 | 1 | 1 | 1 | 1 | 1 |  |  |  |  |  | **** |
| Jemini-Gashi | 2022 | 1 | 1 | 1 | 1 | 1 | 1 | 1 |  |  |  |  |  | **** |
| Jenholt-Nolbris | 2022 | 1 | 1 |  |  |  |  |  | 1 | 1 | 1 | 1 | 1 | **** |
| Kelly | 2021 | 1 | 1 | 1 | 1 | 1 | 1 | 1 |  |  |  |  |  | **** |
| Larcher | 2020 | 1 | 1 | 1 | 1 | 1 | 1 | 1 |  |  |  |  |  | **** |
| Loer | 2022 | 1 | 1 | 1 | 1 | 1 | 1 | 1 |  |  |  |  |  | **** |
| Lukoševičiūtė | 2022 | 1 | 1 | 1 | 1 | 1 | 1 | 1 |  |  |  |  |  | **** |
| Maftei | 2022 | 1 | 1 |  |  |  |  |  | 1 | 1 | 1 | 1 | 1 | **** |
| Malmquist | 2022 | 1 | 1 | 1 | 1 | 1 | 1 | 1 |  |  |  |  |  | **** |
| McCluskey | 2021 | 1 | 1 | 1 | 1 | 1 | 1 | 1 |  |  |  |  |  | **** |
| McKinlay | 2021 | 1 | 1 | 1 | 1 | 1 | 1 | 1 |  |  |  |  |  | **** |
| O'Kane | 2021 | 1 | 1 |  |  |  |  |  | 1 | 1 | 1 | 1 | 1 | **** |
| Olah | 2022 | 1 | 1 | 1 | 1 | 1 | 1 | 1 |  |  |  |  |  | **** |
| Perming | 2022 | 1 | 1 | 1 | 1 | 1 | 1 | 1 |  |  |  |  |  | **** |
| Phillips | 2022 | 1 | 1 | 1 | 1 | Can't tell | 1 | 1 |  |  |  |  |  | *** |
| Procentese | 2021 | 1 | 1 | 1 | 1 | 1 | 1 | 1 |  |  |  |  |  | **** |
| Riiser | 2021 | 1 | 1 | 1 | 1 | 1 | 1 | 1 |  |  |  |  |  | **** |
| Roberts | 2021 | 1 | 1 | 1 | 1 | 1 | 1 | 1 |  |  |  |  |  | **** |
| Sawyer | 2022 | 1 | 1 | 1 | 1 | 1 | 1 | 1 |  |  |  |  |  | **** |
| Scott | 2021 | 1 | 1 | 1 | 1 | 1 | 1 | 1 |  |  |  |  |  | **** |
| Sica | 2022 | 1 | 1 | 1 | 1 | 1 | 1 | 1 |  |  |  |  |  | **** |
| Stewart | 2022 | 1 | 1 |  |  |  |  |  | 1 | 1 | 1 | 1 | 1 | **** |
| Thompson | 2021 | 1 | 1 | 1 | 1 | 1 | 1 | 1 |  |  |  |  |  | **** |
| Tishelman | 2022 | 1 | 1 | 1 | 1 | 1 | 1 | 1 |  |  |  |  |  | **** |
| Torronen | 2022 | 1 | 1 | 1 | 1 | 1 | 1 | 1 |  |  |  |  |  | **** |
| Town | 2021 | 1 | 1 | 1 | 1 | 1 | 1 | 1 |  |  |  |  |  | **** |
| Tse | 2021 | 1 | 1 |  |  |  |  |  | 1 | 1 | 1 | 0 | 0 | ** |
| Widnall | 2022 | 1 | 1 | 1 | 1 | 1 | 1 | 1 |  |  |  |  |  | **** |
| Winter | 2022 | 1 | 1 | 1 | 1 | 1 | 1 | 1 |  |  |  |  |  | **** |
| Wood | 2021 | 1 | 1 | 1 | 1 | 1 | 1 | 1 |  |  |  |  |  | **** |

*meets 25% of MMAT criteria. **meets 50% of MMAT criteria. ***meets 75% of MMAT criteria. ****meets 100% of MMAT criteria. U, Unclassified.

Reference: Pluye, P., Robert, E., Cargo, M., Bartlett, G., O’Cathain, A., Griffiths, F., et al. (2011). Proposal: A Mixed Methods Appraisal Tool for Systematic Mixed Studies Reviews. Available online at: http://mixedmethodsappraisaltoolpublic.pbworks.com (Accessed December 7, 2017).
