# Supplementary material for: The impact of COVID-19 on young people’s mental health, wellbeing and routine from a European perspective: A co-produced qualitative systematic review
Source: PLoS One. 2024 Mar 20;19(3):e0299547. doi: 10.1371/journal.pone.0299547 (PMC10954119; doi:10.1371/journal.pone.0299547)
Supplement: S3 Table — (DOCX) [file pone.0299547.s006.docx]

Supplementary file 6: CERQUAL confidence ratings

| Review finding | Confidence in the  evidence | Explanation of confidence in the evidence assessment |
| --- | --- | --- |
| Social connection | | |
| 1. Managing changes in relationships | Moderate confidence | In general, the studies were moderately well done. This finding was seen across many studies and settings. |
| 1. Social disconnection | Moderate confidence | In general, the studies were moderately well done. This finding was seen across several studies and settings. |
| 1. Strengthening relationships | Moderate confidence | In general, the studies were moderately well done. This finding was seen across several studies and settings. |
| 1. Loneliness | Moderate confidence | In general, the studies were moderately well done. This finding was seen across several studies and settings. |
| 1. Feeling connected and supported | Moderate confidence | In general, the studies were moderately well done. The finding was seen across several studies and settings. |
| Education and learning | | |
| 1. Disruption to education | Moderate confidence | In general, the studies were moderately well done. This finding was seen across several studies and settings. |
| 1. Change to school and learning | Moderate confidence | In general, the studies were moderately well done. This finding was seen across many studies and settings. |
| 1. Education and employment support needed | Moderate confidence | In general, the studies were moderately well done. This finding was seen across several studies and settings. |
| 1. Personal development | Low confidence | In general, the studies were moderately well done. However, the sub-theme only captured studies from Ireland and UK. |
| Pandemic information and restrictions | | |
| 1. Accessing information about the pandemic | Low confidence | In general, the studies were moderately well done. However, the finding was only from two studies in the UK. |
| 1. Guidelines and restrictions | Moderate confidence | In general, the studies were moderately well done. This finding was seen across many studies and settings. |
| 1. Information, transparency and mistrust | Moderate confidence | In general, the studies were moderately well done. This finding was seen across several studies and settings. |
| 1. Overload and desensitisation | Low confidence | In general, the studies were moderately well done. However, the finding was only from two studies in the UK and one in Norway. |
| 1. Safety and compliance | Moderate confidence | In general, the studies were moderately well done. This finding was seen across several studies and settings. |
| 1. Managing information intake | Low confidence | In general, the studies were moderately well done. However, this finding was only from two studies in the UK. |
| Mental health support | | |
| 1. Need for intersectional support | Low confidence | In general, the studies were moderately well done. However, this finding was only from two studies in Scotland. |
| 1. Accessing support to stay well | Low confidence | In general, the studies were moderately well done. However, this finding was only from two studies in the UK. |
| 1. Access to support and services | Moderate confidence | In general, the studies were moderately well done. This finding was seen across several studies and settings. |
| 1. Health impacts long-term | Low confidence | The study was moderately well done. However, this finding was only from one study in the UK. |
| 1. Adjustment and need for digital mental health support | Moderate confidence | In general, the studies were moderately well done. This finding was seen across several studies and settings. |
| 1. Mental health self-management | Moderate confidence | In general, the studies were moderately well done. However, this finding was only from four UK studies. |
| 1. Professional support | Moderate confidence | In general, the studies were moderately well done. This finding was seen across several studies and settings. |
| Emotional, lifestyle and behavioural changes | | |
| 1. Boredom, motivation and sleep | Moderate confidence | In general, the studies were moderately well done. This finding was seen across several studies and settings. |
| 1. Differences in feelings and experiences | Moderate confidence | In general, the studies were moderately well done. This finding was seen across several studies and settings. |
| 1. Stress, anxiety, worry and fear | Moderate confidence | In general, the studies were moderately well done. This finding was seen across many studies and settings. |
| 1. Changing perspectives | Moderate confidence | In general, the studies were moderately well done. This finding was seen across several studies and settings. |
| 1. Positive outlook | Moderate confidence | In general, the studies were moderately well done. This finding was seen across many studies and settings. |
| 1. Homelife and environment | Moderate confidence | In general, the studies were moderately well done. This finding was seen across several studies and settings. |
| 1. Lifestyle behaviour changes | Moderate confidence | In general, the studies were moderately well done. This finding was seen across many studies and settings. |
| 1. Routine and structure | Moderate confidence | In general, the studies were moderately well done. This finding was seen across several studies and settings. |
| 1. Worries about the future | Low confidence | In general, the studies were moderately well done. However, this finding was only from three studies in the UK. |
| 1. Finance changes | Moderate confidence | In general, the studies were moderately well done. However, this finding was only from UK studies. |
| 1. Progressive social, economic and lifestyle changes | Moderate confidence | In general, the studies were moderately well done. However, this finding was only from UK studies. |
| 1. Life on hold | Moderate confidence | In general, the studies were moderately well done. This finding was seen across several studies and settings. |
| 1. A new normal | Moderate confidence | In general, the studies were moderately well done. This finding was seen across several studies and settings. |
| 1. Future challenges | Moderate confidence | In general, the studies were moderately well done. However, this finding was only from Ireland and Great Britain studies. |
| 1. Outside space | Moderate confidence | In general, the studies were moderately well done. However, this finding was only from UK studies. |
| 1. Physical activities | Moderate confidence | In general, the studies were moderately well done. This finding was seen across several studies and settings. |
| 1. Keeping busy | Low confidence | In general, the studies were moderately well done. However, this finding was only from one Ireland and one UK study. |
